# Novel agonist and antagonist radioligands for the GLP‐2 receptor. Useful tools for studies of basic GLP‐2 receptor pharmacology

**DOI:** 10.1111/bph.15766

**Published:** 2022-01-11

**Authors:** Sarina Gadgaard, Wijnand J. C. van der Velden, Sine P. Schiellerup, Jenna Elizabeth Hunt, Maria B. N. Gabe, Johanne Agerlin Windeløv, Geke Aline Boer, Hannelouise Kissow, Cathrine Ørskov, Jens J. Holst, Bolette Hartmann, Mette M. Rosenkilde

**Affiliations:** ^1^ Laboratory of Molecular Pharmacology, Department of Biomedical Sciences, Faculty of Health and Medical Sciences University of Copenhagen Copenhagen Denmark; ^2^ Bainan Biotech Copenhagen Denmark; ^3^ Department of Computational and Quantitative Medicine Beckman Research Institute of the City of Hope Duarte California USA; ^4^ Endocrinology and Metabolism, Department of Biomedical Sciences, Faculty of Health and Medical Sciences University of Copenhagen Copenhagen Denmark; ^5^ Novo Nordisk Foundation Center for Basic Metabolic Research University of Copenhagen Copenhagen Denmark

**Keywords:** association and dissociation rates, class B1 GPCR, GLP‐2 recetor expression, oxidative iodination, radioligands as tool compound, receptor binding kinetics, receptor‐ligand interactions

## Abstract

**Background:**

Glucagon‐like peptide‐2 (GLP‐2) is a pro‐glucagon‐derived hormone secreted from intestinal enteroendocrine L cells with actions on gut and bones. GLP‐2(1–33) is cleaved by DPP‐4, forming GLP‐2(3–33), having low intrinsic activity and competitive antagonism properties at GLP‐2 receptors. We created radioligands based on these two molecules.

**Experimental approach:**

The methionine in position 10 of GLP‐2(1–33) and GLP‐2(3–33) was substituted with tyrosine (M10Y) enabling oxidative iodination, creating [^125^I]‐hGLP‐2(1–33,M10Y) and [^125^I]‐hGLP‐2(3–33,M10Y). Both were characterized by competition binding, on‐and‐off‐rate determination and receptor activation. Receptor expression was determined by target‐tissue autoradiography and immunohistochemistry.

**Key results:**

Both M10Y‐substituted peptides induced cAMP production via the GLP‐2 receptor comparable to the wildtype peptides. GLP‐2(3–33,M10Y) maintained the antagonistic properties of GLP‐2(3–33). However, hGLP‐2(1–33,M10Y) had lower arrestin recruitment than hGLP‐2(1–33). High affinities for the hGLP‐2 receptor were observed using [^125^I]‐hGLP‐2(1–33,M10Y) and [^125^I]‐hGLP‐2(3–33,M10Y) with *K*_*D*_ values of 59.3 and 40.6 nM. The latter (with antagonistic properties) had higher *B*_max_ and faster on and off rates compared to the former (full agonist). Both bound the hGLP‐1 receptor with low affinity (*K*_i_ of 130 and 330 nM, respectively). Autoradiography in wildtype mice revealed strong labelling of subepithelial myofibroblasts, confirmed by immunohistochemistry using a GLP‐2 receptor specific antibody that in turn was confirmed in GLP‐2 receptor knock‐out mice.

**Conclusion and implications:**

Two new radioligands with different binding kinetics, one a full agonist and the other a weak partial agonist with antagonistic properties were developed and subepithelial myofibroblasts identified as a major site for GLP‐2 receptor expression.

**Abbreviations**
COS‐7: monkey fibroblast
KO: knock‐out

**What is already known**

- Need of high‐affinity radioligands for the GLP‐2 receptor.
- GLP‐2 receptor mRNA transcript expressed in both intestinal and extraintestinal tissues.

**That this study adds**

- Description of two GLP‐2 based radioligands with different binding kinetics and dual selectivity.
- GLP‐2 receptor expression at the protein level in intestinal tissue.

**Clinical significance**

- Confirms GLP‐2 receptor GI‐tract protein expression supporting the therapeutic use of GLP‐2 in short‐bowel‐syndrome.
- Different binding kinetics of peptides with different pharmacological properties.

## 1 INTRODUCTION

The gut hormone glucagon‐like peptide‐2 (GLP‐2) is a 33‐long amino acid peptide (GLP‐2(1–33)) that is derived from the pro‐glucagon gene. GLP‐2 is secreted from the enteroendocrine L cells of the small intestine upon nutrient ingestion and rapidly cleaved by ubiquitous protease dipeptidyl peptidase‐4 (DPP‐4), resulting in the degradation product GLP‐2(3‐33) (Thulesen et al., 2002). In mice, administration of GLP‐2(1–33) promotes growth of the small and large intestine (Drucker et al., 1996; Thulesen et al., 2002), stimulates proliferation of the crypt cells, increases nutrient absorption and promotes healing and maintenance of epithelial integrity (Dubé et al., 2006). These intestinotrophic actions of GLP‐2 have been exploited therapeutically with the use of the DPP‐4 resistant GLP‐2 analogue teduglutide (GLP‐2[1–33,A2G]), which since 2012 has been used in the treatment of short bowel syndrome (SBS) in adults (Jeppesen et al., 2001). In addition, a 4 month clinical study showed that GLP‐2(1–33) has an anti‐catabolic effect on the bone tissue by inhibiting bone resorption (measured by the bone marker C‐terminal telopeptide (CTX) (Askov‐Hansen et al., 2013; Gottschalck, Jeppesen, Hartmann, et al., 2008; Gottschalck, Jeppesen, Holst, et al., 2008; Henriksen et al., 2007, 2009).

The metabolite GLP‐2(3–33) has been shown to display low intrinsic activity in cAMP accumulation with an *E*_max_ of 15% of GLP‐2(1–33) and an EC_50_ of ~6 nM, thus acting as a partial agonist of the GLP‐2 receptor (Thulesen et al., 2002). In the same study, it was shown to inhibit the activity of hGLP‐2(1–33), thus also displaying antagonistic properties *in vitro* and *in vivo*. Structurally, GLP‐2 is closely related to the peptide hormones glucagon‐like peptide‐1 (GLP‐1) and gastric inhibitory peptide/glucose‐dependent insulinotropic polypeptide (GIP). GIP (secreted from enteroendocrine K cells) and GLP‐1 (co‐secreted with GLP‐2 from L cells) are important insulinotropic hormones, whereas GLP‐2 is inactive in this respect (Schiellerup et al., 2019). GLP‐1 analogues are widely used as treatment for type 2 diabetes mellitus and obesity, and more recently, a dual‐agonist of GLP‐1 and GIP showed promising effects within this field (Coskun et al., 2018).

The GLP‐2 receptor is a G protein‐coupled receptor (GPCR), belonging to the subclass B1 of the GPCR family, which comprises 15 receptors including the GLP‐1 receptor the GIP receptor, the glucagon receptor, the secretin receptor and the vasoactive intestinal peptide 1 and 2 receptors (VPAC_1_ and VPAC_2_ ) (Fredriksson et al., 2003). High resolution structures of class B1 GPCRs combined with mutation studies have enabled the analysis of the active, intermediate and inactive conformations of the receptors, thereby revealing residues that are essential for ligand binding and/or activation (Smit et al., 2021; Sun et al., 2020; Zhang et al., 2017).

For many years, the leading paradigm regarding ligand binding to class B1 GPCRs was the ‘two‐step’ binding mechanism, suggesting that the C‐terminus of the peptide ligand switches between an overall disordered and a more ordered alpha‐helical secondary structure. The receptor recognizes and binds the ordered conformation of the peptide ligand, which initiates receptor changes and activation (Parthier et al., 2007). Today, this view has been expanded to include a complex network of conformational changes that takes place upon receptor activation (Liang, Khoshouei, Deganutti, et al., 2018; Venneti & Hewage, 2011; Wu et al., 2020; Zhang et al., 2017). Signalling through class B1 receptors, including the GLP‐2 receptor, mainly occurs through Gα_s_ coupling, thereby evoking multiple signalling cascades, including increased levels of the downstream second messenger cyclic adenosine monophosphate (cAMP). Furthermore, by immunofluorescence microscopy, Estall et al. (2004, 2005) showed that the C‐terminus of the GLP‐2 receptor recruits β‐arrestin‐2 following agonist stimulation but that this recruitment is not required for desensitization or receptor endocytosis of the GLP‐2 receptor. Functional consequences of β‐arrestin recruitment by the GLP‐2 receptor have not yet been described, although important effects hereof have been demonstrated for other class B1 GPCRs, such as the GIP receptor (Gabe et al., 2018) and GLP‐1 receptor (van der Velden et al., 2021).

Although being cloned in 1999, the precise tissue and cellular localization of GLP‐2 receptor expression remains controversial. Messenger RNA (mRNA) transcripts of the GLP‐2 receptor are found within gastro‐intestinal tissues (stomach, duodenum, jejunum, ileum, colon and intestinal ganglion cells) of various species, including human and rodents (Bjerknes & Cheng, 2001; El‐Jamal et al., 2014; Ørskov et al., 2005; Pedersen et al., 2015; Yusta et al., 2000; Yusta et al., 2019) and in the intestinal subepithelial myofibroblasts (SEMF) cell line, CCD‐19Co (El‐Jamal et al., 2014). Further, mRNA transcripts of the GLP‐2 receptor have been reported in various extraintestinal tissues (fat, lymph nodes, bladder, spleen, liver and hepatocytes cells) (El‐Jamal et al., 2014; Yusta et al., 2000) including human and rat pancreas (de Heer et al., 2007), a tissue known for high expression levels of the GLP‐1 receptor (Richards et al., 2014).

In the present study, we investigated two novel radioligands with tyrosine (Tyr)‐substitution at position 10 with methionine (Met) (referred to as M10Y) in the two naturally occurring human GLP‐2 (hGLP‐2) peptides, the agonist GLP‐2(1–33) and its metabolite GLP‐2(3–33), the partial agonist/antagonist. With these, we determined differential binding kinetics *in vitro*. We performed autoradiography studies in mice and hereby showed GLP‐2 receptor protein in the GI tract. With a GLP‐2 receptor specific antibody we confirmed this expression in wildtype (WT) mice, whereas similar gut tissue from GLP‐2 receptor knock‐out (KO) mice displayed no antibody binding.

## 2 METHODS

### 2.1 Cell culturing, transfection and generating of stable GLP‐2 receptor expressing cells

COS‐7 cells (NCBI_Iran Cat# C143, RRID:CVCL_0224) were cultured at 10% CO_2_, 95% air humidity and 37°C in Dulbecco's Modified Eagle Medium (DMEM) 1885 supplemented with 10% foetal bovine serum (FBS), 1% penicillin (180 U ml^−1^)/streptomycin (45 μg·ml^−1^). HEK‐293 cells (CLS Cat# 300192/p777_HEK293, V79020) were cultured in Dulbacco's Modified Eagle Medium (DMEM) containing 1% GlutaMAS supplemented with 10% foetal bovine serum (FBS) and 1% penicillin (180 U ml^−1^)/streptomycin (45 μg·ml^−1^) and incubated at 37°C, 10% CO_2_, 95% air humidity. The cells were transfected using the calcium phosphate precipitation method as previously described (Jensen et al., 2007). Briefly, the cells were seeded in T175/T75/T25 flasks 1 day before transfection with 40/20/10 μg receptor DNA or pcDNA3.1(+) (control). Transiently transfected COS‐7 cells were used in cAMP accumulation and for whole cell homologous and heterologous binding, while transiently transfected HEK‐293 cells were used for β‐arrestin recruitment. HEK‐293 cells stably expressing hGLP‐2 receptor or pcDNA3.1(+) (control) were generated by transfection as described above. The cells were cultured at 10% CO_2_, 95% air humidity and 37°C in Dulbecco's Modified Eagle Medium (DMEM), containing 1% GlutaMAX, and supplemented with 10% foetal bovine serum (FBS) 1% penicillin (180 U ml^−1^)/streptomycin (45 μg·ml^−1^) and 0.4 mg·ml^−1^ G418 for selection. Stably transfected HEK‐293 cells were used for membrane preparation and the corresponding kinetic binding experiments (van der Velden et al., 2021).

### 2.2 Membrane preparation for kinetic binding experiments

The HEK‐293 cells stably expressing hGLP‐2 receptor or pcDNA3.1(+) (control) were harvested using ice cold PBS (supplemented with a cOmplete EDTA free protease inhibitor [Roche, Basel, Switzerland]) and a cell scraper, after which they were homogenized using a Dounce homogenizer. The homogenate was centrifuged for 3 min at 500 rpm (54  g; 4°C), after which the supernatant was centrifuged for 45 min at 14,500 rpm at (24,446 g; 4°C). The resulting pellet was suspended in storage buffer (20 mM HEPES buffer [pH 7.2], 0.4 mM CaCl_2_, 2 mM MgCl_2_ and cOmplete EDTA free protease inhibitor) and stored at −80°C. Protein determination was performed according to a standard Pierce BCA protein assay protocol (Thermo Scientific, Rockford, IL).

### 2.3 cAMP accumulation

For the cAMP measurements, COS‐7 cells were transfected with receptor plasmid or pcDNA3.1(+) (control) and seeded with 25,000 cells per well in a CulturPlate‐96 (PerkinElmer; Waltham, MA) 1 day after transfection. The following day, the cells were washed once with HEPES‐buffered saline and incubated for 30 min at 37°C with HEPES‐buffered saline supplemented with 1 mmol·L^−^ ^1^ 3‐isobutyl‐1‐methylxanthine (IBMX) buffer. To test for intrinsic activity, endogenous hGLP‐2(1–33) or hGLP‐2(3–33) or the M10Y‐substituted variants (hGLP‐2(1–33,M10Y) or hGLP‐2(3–33,M10Y)) were added in increasing concentrations, and the plates were incubated for additional 30 min at 37°C. To test hGLP‐2(3–33) and hGLP‐2(3–33,M10Y) as antagonists the cells were preincubated for 10 min with a fixed concentration of antagonist followed by the addition of increasing concentrations of agonist. Afterwards, the HitHunter cAMP XS‐assay (DiscoverX, Birmingham, UK) was carried out according to the manufacturer's instructions. Luminescence was measured by a Perkin Elmer EnVision 2104 Multilabel reader (PerkinElmer; Waltham, MA).

### 2.4 β‐arrestin recruitment

For β‐arrestin recruitment, 1 × 10^6^ HEK‐293 cells were seeded in 60 mm EasYDish TC surface dishes (ThermoFisher Scientific, Denmark) 1 day prior to transfection. The cells were transiently transfected with 0.66 μg receptor DNA, 0.07 μg Rlus‐8‐arrestin 2 (β‐arrestin 1) or 3 (β‐arrestin 2) and 1.4 μg Membrane‐Citrine‐SH3. The transfected cells were washed with 2 ml PBS and resuspended in 2 ml PBS supplemented with 1% Glucose 48 h after transfection and aliquoted into 96 well plates. To test intrinsic activity hGLP‐2(1–33) or hGLP‐2(3–33) or the M10Y‐substituted variants (hGLP‐2(1–33,M10Y) or hGLP‐2(3–33,M10Y)) were added in increasing concentrations, and arrestin recruitment was measured 40 min after ligand addition. Bioluminescence was measured by a Perkin Elmer EnVision 2104 Multilabel reader (PerkinElmer; Waltham, MA).

### 2.5 Oxidative iodination

The radioligands were created by oxidative iodination with the oxidizing agent ChloramineT. Here the iodine isotope [^125^I] becomes incorporated in the Tyr residue at position 10 of hGLP‐2(1–33,M10Y) or hGLP‐2(3–33,M10Y). Two nmol peptide was dissolved in 10 μl iodination buffer (100 mM phosphate buffer) and 0.4 nCi Na^125^I was added. To limit di‐iodination, a stepwise stoichiometric oxidation reaction was performed by sequential addition of 6 aliquots of 5 μl ChloraminT (30 μg·ml^−1^) with 1 min intervals during constant stirring. Under these conditions, [^125^I] is incorporated at the hydroxyl group in the ortho position of the Tyr residue. The reaction was terminated by the addition of 400 μl phase A (0,1% trifluoracetic acid). The reaction was carried out in at pH 7.4 to avoid labelling of histidine residues at basic conditions (pH > 8.5). The product was fractionated on a high‐performance liquid chromatography (HPLC) (Åkta, GE Healthcare, Boston US) with a C18 column for reverse‐phase (RP) HPCL (RP‐HPCL). The column was initially flushed with 80% phase A and 20% phase B (acetonitrile + 0,1% trifluoracetic acid), and terminally by applying increasing concentrations of phase B. The pressure of the RP‐HPCL was kept constant at 8 MPa with a flow of 1 ml·min^−1^. Before binding assays were performed, the eluted fractions were tested in homologous competition binding (see next section for method).

### 2.6 Homologous and heterologous binding

For the competition binding experiments, COS‐7 cells were transfected with receptor plasmid or pcDNA3.1(+) (control) and seeded with 150,000 cells per well in CulturPlate‐24 (PerkinElmer; Waltham, MA) 1 day after transfection. The number of cells seeded per well was selected to obtain 5–10% specific binding of the radioligands. Day 2 after transfection, the cells were washed twice in prechilled binding buffer (50 mM HEPES buffer, supplemented with 1 mM CaCl_2_, 5 mM MgCl_2_ and 0,5% (w/v) Bovine serum albumine (BSA)) at pH 8 and incubated for 15 min at 4°C. Increasing concentration of unlabelled ligand followed by a low (13–86 pM) concentration of the radioligand (21,400–30,000 cpm/well) were added to the cells, which were then incubated for additional 3 h at 4°C. After incubation, the cells were washed twice in prechilled binding buffer, lysed and counted using a Wizard gamma counter (PerkinElmer; Waltham, MA).

### 2.7 Kinetic binding experiments

The association assays were performed by preparing a mixture of 10 μg membranes from HEK‐293 cells stably expressing hGLP‐2 receptor or pcDNA3.1(+) (control) and 0.5 mg wheatgerm agglutinin coated (WGA) PVT SPA beads (PerkinElmer; Waltham, MA). The decay particles from [^125^I] will stimulate the scintillant within the SPA beads to emit light when it is in proximity. This mixture was pre‐coupled on a shaker in a total volume of 50 μl binding buffer (50 mM HEPES buffer [pH 8.0]), supplemented with 1 mM CaCl_2_, 5 mM MgCl_2_ and 0,5% (w/v) BSA) for 30 min at 30°C. The pre‐coupling was followed by the distribution of membrane suspension in a CulturPlate‐96 (PerkinElmer; Waltham, MA) in a total volume of 90 μl binding buffer and spun down afterwards (1,500 RPM, 485 g, 5 min, room temperature). The reaction was initiated by the addition of 0.19 ± 0.001 nM [^125^I]‐hGLP‐2(1–33,M10Y) or 0.21 ± 0.004 nM [^125^I]‐hGLP‐2(3–33,M10Y) (10 μl), and the amount of radioligand bound to receptor was measured every minute up to 100 min [^125^I]‐hGLP‐2(3–33,M10Y) or 120 min [^125^I]‐hGLP‐2(1–33,M10Y) at 30°C, using a TopCount NXT Microplate Scintillation and Luminescence Counter (PerkinElmer; Waltham, MA). The specific binding of each radioligand to hGLP‐2 receptor was calculated by measuring the total binding (membranes from HEK‐293 cells expressing hGLP‐2 receptor) and the non‐specific binding (HEK‐293 cell membranes expressing pcDNA3.1(+)) at each time‐point (specific binding = total binding minus non‐specific binding).

For the dissociation experiments, the membrane suspension was added to the wells in a total volume of 85 μl binding buffer. The mixture was then pre‐incubated for 60 min at 30°C with 0.19 ± 0.001 nM [^125^I]‐hGLP‐2(1–33,M10Y) or 0.21 ± 0.004 nM [^125^I]‐hGLP‐2(3–33,M10Y) (10 μl). The dissociation was initiated by the addition of 5 μl of 1 μM unlabelled hGLP‐2(1–33,M10Y) or hGLP‐2(3–33,M10Y). The amount of bound radioligand was measured every minute up to 500 min.

### 2.8 Immunohistology and autoradiography

All procedures using mice were approved by the Danish National Animal Experiments Inspectorate (licence no. 2018‐15‐0201‐01397) and repported in compliance with the ARRIVE guidelines (Percie du Sert et al., 2020) and with the recommendations made by the *British Journal of Pharmacology* (Lilley et al., 2020). All mice were kept in the animal facility and received tap water and standard chow diet (altromin 1314, Altromin Spezialfutter GmbH and Co, Germany) *ad libitum*.

#### 2.8.1 Surgical procedure

Female C57BL/6JRj (MGI Cat# 5752053, RRID:MGI:5752053) mice (*n* = 9) weighing 18–26 g (age 9–12 weeks) were purchased from Janvier (Saint Berthevin Cedex, France). All mice were housed in Type III conventional cages with up to 8 mice per cage (Tecniplast, Via I Maggio, Buguggiate [VA]) and left to acclimatize for at least 1 week before experimental procedures. The mice were anaesthetized with an intraperitoneal injection of ketamine‐xylazine (100:10 mg·kg^−1^, Pharma service SUND, UCPH, Copenhagen, Denmark). The abdomen was opened by a midline incision, the inferior caval vein exposed and [^125^I]‐hGLP‐2(1–33,M10Y) (2–3 pmol [3–5 mill cpm]) dissolved in 100 μl 0.04 M phosphate buffer containing 1% HSA (pH 7.5) was injected. Half of the animals also received >10,000‐fold excess of unlabelled hGLP‐2(1–33,M10Y) (30 nmol) in combination with the labelled peptide in the same injection to test for specific binding. The actual amount of [^125^I]‐labelled peptide administered was calculated from the specific radioactivity of the radioligand. Before injection, 10 μl of the [^125^I]‐labelled peptide stock solution was counted in a gamma‐counter to determine the amount of radioactivity injected into the animals. Ten minutes after peptide injection, the thorax was opened, after which the vascular system was perfused at a constant flow with 0.9% saline with an outlet through the right ventricle. The body temperature of the animals during the precedure were maintained using a heat lamp while no breating assistence were provided. Next, the mice were fixated by flushing the system with ice‐cold 4% paraformaldehyde. After fixation, the pancreas, small intestine and kidneys (as positive control) were removed and stored in 45% paraformaldehyde until further processing.

#### 2.8.2 Autoradiography

Small intestinal, pancreatic and kidney tissue samples were embedded in paraffin, and histological 4 μm sections were cut with a microtome and placed on glass slides. The sections were dewaxed and coated in a dark room with 43–45°C Kodak NTB emulsion (VWR, Herlev, Denmark) diluted 1:1 with 43–45°C water and subsequently dried and stored in light‐proof boxes at 5°C for 6 weeks. After 6 weeks, the tissue sections were developed in a dark room in Kodak D‐19 developer (VWR, Herlev, Denmark) for 5 min, dipped 10 times in 0.5% acetic acid and fixated in 30% sodium thiosulphate for 10 min. The sections were then washed, first in water for 10 min and then in 70% ethanol. Finally, the sections were lightly counterstained with haematoxylin and examined with a light microscope (Orthoplan, leitz). Images were taken with an AxioCam ICc5 camera (Zeiss) connected to the light microscope.

#### 2.8.3 Immunohistochemistry

The Immuno‐related procedures used comply with the recommendations made by the *British Journal of Pharmacology* (Alexander et al., 2018). Specimens of pancreas and small intestine (*n* = 5) from wild‐type mice and GLP‐2 receptor KO mice were fixed in formalin buffer 10% and embedded in paraffin. The tissue blocks were cut in sections of 4 μM and dewaxed through xylene to tap water.

For antigen retrieval, the sections were boiled in a microwave oven for 15 min in a 10 mmol Tris‐EDTA‐buffer pH 9 followed by pre‐incubation in 2% BSA for 10 min and an overnight incubation at 4°C with the polyclonal primary rabbit GLP‐2 receptor antibody 99,077 diluted 1:16,000 in PBS containing in addition 2% BSA (Ørskov et al., 2005). On day 2, the sections were washed and incubated with biotinylated secondary goat‐anti rabbit antibody (Vector Laboratories, Cat# BA‐1000, RRID:AB_2313606) diluted 1:200. Three per cent hydrogenperoxide was added to the tissue slides to block endogenous peroxidase activity followed by a 30 min incubation of Avidin and Biotinylated horseradish peroxidase to form macromolecular Complexes (Code nr. PK‐6100, Vector Laboratories, ABC‐Elite). Finally, the reaction was developed with 3,3–diaminobenzidine (DAB ImmPACT, SK‐4105, Vector Laboratories) for 15 min and counterstaining using Mayers Hæmatoxylin.

### 2.9 Design of the experiments and statistical analysis

Studies were designed to generate groups of equal sizes. In some cases, the group size for the *in vitro* experiments was below 5 as sufficient data were obtained with less experiments. For *in vivo* autoradiography and immunohistochemistry, five animals were included in each group. Due to the three Rs and the loss of one animal in each group, only four animals per group are shown. For these data, no statistical analysis was conducted. Special randomization and blinded analysis were not necessary for the *in vitro* studies because the cells used in the experiments could be maintained under the same experimental conditions. To avoid experimental bias, the same experiment was not repeated more than twice a week. The data and statistical analysis comply with the British Journal of Pharmacology on experimental design and analysis in pharmacology (Curtis et al., 2018). Statistical analysis was only carried out for data with a group size of *n* ≥ 5 for each group. Statistical significances between dose–response curves and *B*_max_ were analysed using paired Student's *t*‐test with a level of significance of 0.05. The group size, *n*, value represents the number of independent experiments, and statistical analysis was carried out using independent values. To compensate for inter‐assay variations, the data were normalized to the binding of reference radioligand or activation by the endogenous full agonist (see figure legends for further explanation in each assay). All experimental data points were included in the data analysis. The analysis was interpreted using GraphPad Prism 8.0 software (Graphpad software, RRID:SCR_002798) to obtain the following parameters: IC_50_, EC_50_, *E*_max_, *k*_on_, *k*_off_, *k*_obs_ and *B*_max_. All sigmoidal curves were fitted with a Hill slope of either 1 for activation curves or −1 for inhibition curves. All data were expressed as mean ± standard error of the mean (SEM), if not otherwise stated.

### 2.10 Data analysis

*B*_max_ (the total density of receptors in the sample) were calculated from homologous competitive binding curves according to Equation 1 (Richards et al., 2014):

(1) $$B_{\max}=\frac{B_{0}\cdot {\mathrm{IC}}_{50}}{\left[L\right]},$$

where *B*_0_ is the total specific binding in CPM and [*L*] is the concentration of radioligand in nM.

The equilibrium dissociation constant (*K*_*D*_) was also calculated from the homologous competitive binding curves according to Equation 2:

(2) $$K_{D}={\mathrm{IC}}_{50}-\left[L\right].$$

The inhibition constant (*K*_i_) was obtained from the heterologous competitive binding curves by using the Cheng and Prusoff equation according to Equation 3 (Cheng & Prusoff, 1973):

(3) $$K_{i}=\frac{{\mathrm{IC}}_{50}}{1+\left(\frac{\left[L\right]}{K_{D}}\right)}.$$

The association rate constant (*k*_on_) was calculated according to Equation 4 (van der Velden et al., 2020):

(4) $$k_{\mathrm{on}}=\frac{k_{\mathrm{obs}}-k_{\mathrm{off}}}{\left[L\right]},$$

where *k*_obs_ is the observed association rate constant (min^−1^) and *k*_off_ is the dissociation rate constant (min^−1^).

The *K*_*D*_ calculated from the kinetic parameters was calculated according to Equation 5:

(5) $$K_{D}=\frac{k_{\mathrm{off}}}{k_{\mathrm{on}}},$$

where *k*_off_ and *k*_on_ are determined from the membrane binding.

The BRET signal was obtained by calculating the BRET ration using Equation 6:

(6) $$\text{BRET}=\frac{\mathrm{YFP}}{\text{RLUC}}.$$

### 2.11 Bioinformatics

Sequence similarities (%) were evaluated by protein blast at the National Center for Biotechnology Information (NCBI, RRID:SCR_006472). The amino acid alignment was acquired my Multiple Sequence Alignment using the Clustal Omega software available at the EMBL‐EBI website (Clustal Omega, RRID:SCR_001591).

### 2.12 Materials

pcDNA3.1(+) plasmids encoding the human, rat, mouse GLP‐2 or GLP‐1 receptors were obtained from ThermoFisher (ThermoFisher Scientific, Denmark, cat# V79020, pcDNA™3.1(+) Mammalian Expression Vector). All ligand peptides were purchased from CASLO ApS (Technical University of Denmark, DTU‐Science Park) with a minimum purity of 95%. Na^125^I with specific activity of ~17 Ci/mg (pH 8–11) was obtained from Perkin Elmer (Waltham, Massachusetts, US). The plasmids for arrestin recruitment (Rluc‐Arrestin 2/3 and Membrane‐Citrone‐SH3) were kindly provided by Jonathan A. Javitch, Columbia University.

### 2.13 Nomenclature of targets and ligands

Key protein targets and ligands in this article are hyperlinked to corresponding entries in the IUPHAR/BPS Guide to PHARMACOLOGY (http://www.guidetopharmacology.org) and are permanently archived in the Concise Guide to PHARMACOLOGY 2020/21 (Alexander et al., 2021).

## 3 RESULTS

### 3.1 GLP‐2(1–33) with Tyr in position 10 displays strong cAMP accumulation but impaired β‐arrestin recruitment

**FIGURE 1:**
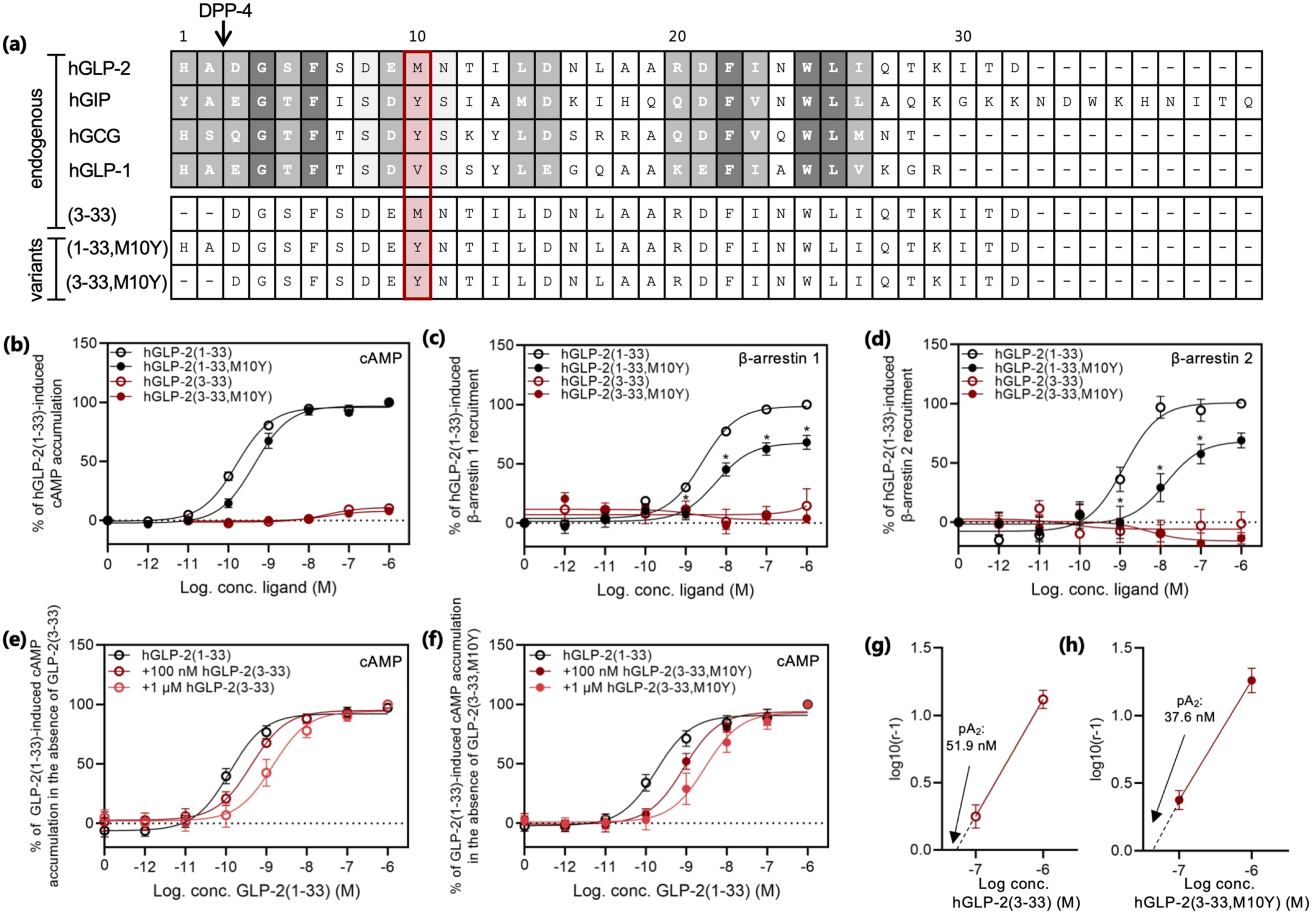
Sequence alignment of GLP‐2 and related peptides and activity of hGLP‐2 and variants at the hGLP‐2 receptor. (a) Alignment of the class B1 GPCR peptides; hGLP‐2(1–33), hGIP(1–42), hglucagon (GCG)(1–29), hGLP‐1(7–36) (top panel) and the GLP‐2 variants; hGLP‐2(3–33), hGLP‐2(1–33,M10Y) and hGLP‐2(3–33,M10Y) (bottom panel). Dark grey refers to positions, which are fully conserved (identical); medium grey refers to positions with strongly similar residues, while light grey refers to positions with weakly similar residues. The red box marks position 10 (counted from residue 1 of hGLP‐2(1–33)). Dose–response curve in (b) cAMP accumulation, (c) β‐arrestin 1 recruitment and (d) β‐arrestin 2 recruitment for the hGLP‐2 receptor stimulated with increasing concentrations of hGLP‐2(1–33) and, hGLP‐2(1–33,M10Y), both in black, and, hGLP‐2(3–33), and hGLP‐2(3–33,M10Y), both in red. cAMP accumulation dose–response curve for hGLP‐2(1–33) in the presence of 100 nM and 1 μM (e) hGLP‐2(3–33) or (f) hGLP‐2(3–33,M10Y), and the corresponding Schild plots shown in (g) hGLP‐2(3–33) and (h) hGLP‐2(3–33,M10Y) with pA_2_‐values. To compensate for inter‐assay variations data were normalized to 1 µM hGLP‐2(1–33)‐mediated activation and recruitment within each assay. Potencies, efficacies and number of experiments are included in Table 1

Native hGLP‐2 does not contain a Tyr residue and is therefore unsuitable for oxidative iodination. Many residues are conserved among class B1 ligand‐receptor pairs and high sequence similarities are found between GLP‐2 and the other class B1 hormones hGIP, hglucagon and hGLP‐1 (Figure 1a). This enabled us to look for a suitable position for introduction of a Tyr residue and for [^125^I]‐labelling of hGLP‐2. Position 10 of this ligand class is not fully conserved, meaning that whereas hGIP and glucagon have a Tyr residue at this position, a Met residue is found in GLP‐2. In hGIP and glucagon, this Tyr residue serves as the backbone for oxidative [^125^I]‐labelling (Kuc et al., 2014; Pingoud, 1985). We therefore substituted Met in position 10 of GLP‐2 with a Tyr residue in order to target it for oxidative iodination (Figure 1a). Because GLP‐2(1–33) is rapidly cleaved to hGLP‐2(3–33) which has decreased intrinsic activity (*E*_max_ of 15%) and antagonistic properties (Thulesen et al., 2002), we modified both peptides to create the two concordant peptides (hGLP‐2(1‐33,M10Y) and (hGLP‐2(3‐33,M10Y)) with the intension to create two radioligands with different pharmacodynamics properties.

First, we measured the ligand‐mediated receptor activation of the two altered peptides in terms of cAMP accumulation. COS‐7 cells, transiently expressing hGLP‐2 receptor, were stimulated with increasing concentrations of the two GLP‐2 variants and compared to the corresponding endogenous GLP‐2 peptides. The hGLP‐2(3–33) accumulated cAMP with an 11% efficacy compared to the full agonist hGLP‐2(1–33) (Figure 1b and Table 1), consistent with previously shown data (Thulesen et al., 2002). In contrast, hGLP‐2(1–33,M10Y) displayed strong and full receptor activation with only a 2.8‐fold decreased potency compared to hGLP‐2(1–33) (Figure 1b and Table 1). Similar to the endogenous metabolite hGLP‐2(3–33), hGLP‐2(3–33,M10Y) activated the GLP‐2 receptor with nanomolar potency and low efficacy (Figure 1b and Table 1).

**TABLE 1:** Functional characterization—cAMP accumulation and arrestin recruitment

	Ligand	pEC_50_ (M) ± SEM	EC_50_ (nM)	Efficacy ± SEM	n
hGLP‐2R	**cAMP accumulation**				
	hGLP‐2(1–33)	9.8 ± 0.05	0.16	100 ± 2.5	11
	hGLP‐2(1–33,M10Y)	9.4 ± 0.07	0.44	96 ± 2.9	5
	hGLP‐2(3–33)	7.6 ± 0.28	27.5	11 ± 1.5	3
	hGLP‐2(3–33,M10Y)	7.5 ± 0.32	31.6	8.1 ± 1.2	7
	hGLP‐2 + 100 nM hGLP‐2(3–33)	9.4 ± 0.10	0.42	95.0 ± 2.9	5
	hGLP‐2 + 1 μM hGLP‐2(3–33)	8.8 ± 0.15	1.44	94.3 ± 4.7	5
	hGLP‐2 + 100 nM hGLP‐2(3–33,M10Y)	9.1 ± 0.10	0.87	93.8 ± 3.1	5
	hGLP‐2 + 1 μM hGLP‐2(3–33,M10Y)	8.5 ± 0.16	2.97	93.3 ± 5.1	5
	**β‐Arrestin 1 recruitment**				
	hGLP‐2(1–33)	8.6 ± 0.05	2.6	98.6 ± 1.8	8
	hGLP‐2(1–33,M10Y)	8.2 ± 0.15	5.7	67.7 ± 3.1	5
	hGLP‐2(3–33)	*N.A*.	*N.A*.	*N.A*.	5
	hGLP‐2(3–33,M10Y)	*N.A*.	*N.A*.	*N.A*.	5
	**β‐Arrestin 2 recruitment**				
	hGLP‐2(1–33)	8.9 ± 0.13	1.2	100.8 ± 4.9	6
	hGLP‐2(1–33,M10Y)	7.8 ± 0.27	14.0	68.5 ± 8.2	6
	hGLP‐2(3–33)	*N.A*.	*N.A*.	*N.A*.	6
	hGLP‐2(3–33,M10Y)	*N.A*.	*N.A*.	*N.A*.	6
hGLP‐1R	**cAMP accumulation**				
	hGLP‐1	10.5 ± 0.04	0.03	100 ± 1.3	9
	hGLP‐2(1–33)	7.0 ± 0.12	98	88 ± 6.0	7
	hGLP‐2(1–33,M10Y)	7.0 ± 0.13	112	100 ± 8.5	3
	hGLP‐2(3–33)	*N.A*.	*N.A*.	*N.A*.	6
	hGLP‐2(3–33,M10Y)	*N.A*.	*N.A*.	*N.A*.	6
	hGLP‐2 + 100 nM exedin(9–39)	6.1 ± 0.83	881	31 ± 8,9	5
	hGLP‐1 + 1 μM hGLP‐2(3–33)	10.4 ± 0.08	0.04	93.2 ± 2.0	5
	hGLP‐1 + 100 nM hGLP‐2(3–33)	10.1 ± 0.11	0.08	95.8 ± 3.2	5
	hGLP‐1 + 1 μM hGLP‐2(3–33,M10Y)	10.5 ± 0.08	0.03	100.1 ± 2.0	5
	hGLP‐1 + 100 nM hGLP‐2(3–33,M10Y)	10.1 ± 0.10	0.07	98.8 ± 2.7	5
	**β‐Arrestin 1 recruitment**				
	hGLP‐1(7–36)	8.6 ± 0.06	0.41	*104.3* ± 2.1	8
	hGLP‐2(1–33)	*N.A*.	*N.A*.	*N.A*.	8
	hGLP‐2(1–33,M10Y)	*N.A*.	*N.A*.	*N.A*.	8
	hGLP‐2(3–33)	*N.A*.	*N.A*.	*N.A*.	8
	hGLP‐2(3–33,M10Y)	*N.A*.	*N.A*.	*N.A*.	8
	**β‐Arrestin 2 recruitment**				
	hGLP‐1(7–36)	8.6 ± 0.06	0.36	105.7 ± 2.3	8
	hGLP‐2(1–33)	*N.A*.	*N.A*.	*N.A*.	8
	hGLP‐2(1–33,M10Y)	*N.A*.	*N.A*.	*N.A*.	8
	hGLP‐2(3–33)	*N.A*.	*N.A*.	*N.A*.	8
	hGLP‐2(3–33,M10Y)	*N.A*.	*N.A*.	*N.A*.	8

*Note*: cAMP accumulation and arrestin recruitment profiles of the four GLP‐2‐based ligands at the GLP‐2 receptor (R) and the GLP‐1 receptor. Potency and efficacy of GLP‐2, GLP‐2 variants and GLP‐1 in cAMP accumulation and β‐arrestin recruitment at the human GLP‐2 receptor and the human GLP‐1 receptor. All data were fitted with a three‐parameter logistic curve to obtain pEC_50_. Data represent the mean ± SEM of at least three experiments performed in duplicates. The numbers of independent experiments, *n*, are indicated in the right column. N.A. refers to no activation detected as saturation were not obtained (*E*_max_ ± SEM) at 10 nM hGLP‐2(1–33).

Next, as an additional functional readout for the hGLP‐2 receptor, we measured β‐arrestin 1 and 2 recruitment in response to the four peptides (Figure 1c,d and Table 1). While the endogenous hGLP‐2(1–33) displayed strong recruitment of both β‐arrestin 1 and 2 with similar potencies, hGLP‐2(1–33,M10Y) displayed lower efficacy and decreased potency (2.2‐ and 11.7‐fold, respectively), thereby demonstrating partial agonism of the M10Y substituted variant of hGLP‐2(1–33) (Figure 1c,d and Table 1). No recruitment of β‐arrestin 1 and 2 was observed for hGLP‐2(3–33) or hGLP‐2(3–33,M10Y). Together, these data demonstrate that the M10Y substitution affects the arrestin recruitment of GLP‐2 receptor more drastically than the cAMP accumulation.

As GLP‐2(3–33) has previously been described as a competitive antagonist for the hGLP‐2 receptor (Thulesen et al., 2002), we determined the antagonistic properties of hGLP‐2(3–33) and hGLP‐2(3–33,M10Y) in the presence of the endogenous agonist in the cAMP accumulation assay. Increasing concentrations (100 nM and 1 μM) of both N‐terminally truncated variants resulted in a rightward shift of the done‐response curve of hGLP‐2(1–33) (Figure 1e,f and Table 1). Thus, the EC_50_ of hGLP‐2(1–33) increased with 3.4‐ and 11.6‐fold in the presence of 100 nM and 1 μM of hGLP‐2(3–33), respectively, and 4.8‐ and 16.3‐fold in the presence of similar doses of hGLP‐2(3–33,M10Y). Schild plot analysis revealed that both acted as competitive antagonists of G protein‐mediated signalling with a Hill slope of 1.15 ± 0.11 and 1.13 ± 0.11 for hGLP‐2(3–33) and hGLP‐2(3–33,M10Y), respectively, and with pA2 values of 51.9 and 37.6 nM, respectively (Figure 1f,g). These data demonstrate that the two N‐terminally truncated GLP‐2 variants act as competitive antagonists in the presence of the endogenous agonist, and alone display weak intrinsic activity and thereby partial agonistic properties in the cAMP accumulation assay.

### 3.2 Higher *B*_max_ and faster on‐ and off‐rate of the radioligand based on hGLP‐2(3–33,M10Y) compared to that based on hGLP‐2(1–33,M10Y)

**FIGURE 2:**
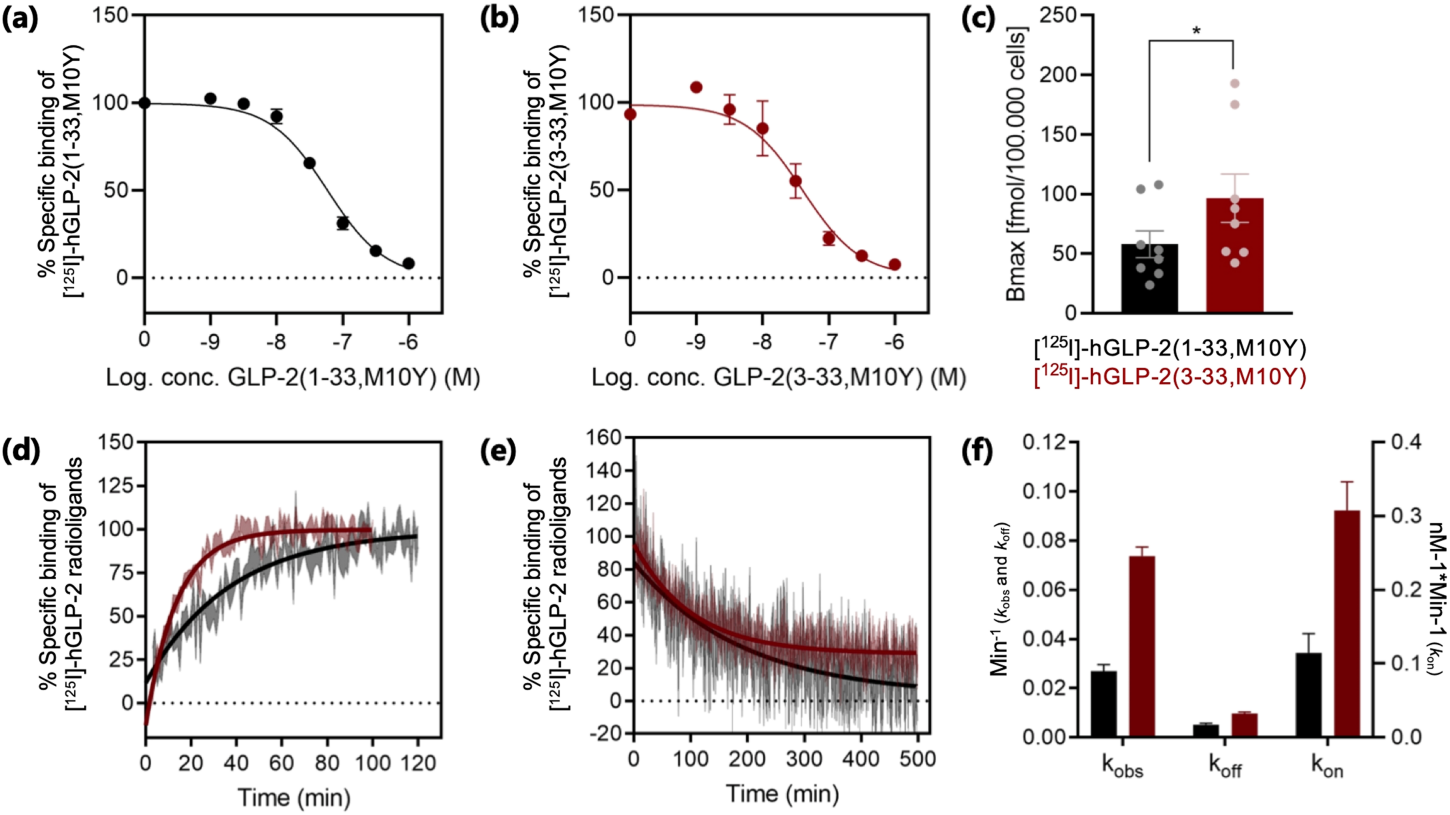
Homologous competition binding and binding kinetic experiments. Homologous competition binding using (a) [^125^I]‐hGLP‐2(1–33,M10Y) (black) (*n =* 5) and (b) [^125^I]‐hGLP‐2(3–33,M10Y) (red) (*n* = 5). To compensate for inter‐assay variations, data were normalized to the specific binding to hGLP‐2 receptor within each assay. (c) *B*_max_ for [^125^I]‐hGLP‐2(1–33,M10Y) (black) and [^125^I]‐hGLP‐2(3–33,M10Y) (red), (d) association (*n* = 4) and (e) dissociation (*n* = 4) of [^125^I]‐hGLP‐2(1–33,M10Y) (black) and [^125^I]‐hGLP‐2(3–33,M10Y) (red) on/from the hGLP‐2 receptor. The dissociation was initiated by the addition of 1 μM unlabelled hGLP‐2(1–33,M10Y) or hGLP‐2(3–33,M10Y). (f) Comparison of binding kinetic parameters between [^125^I]‐hGLP‐2(1–33,M10Y) (black) and [^125^I]‐hGLP‐2(3–33,M10Y) (red) obtained from association and dissociation assays. The parameters *k*_on_, *k*_off_, *k*_obs_ and *K*_*D*_ were calculated from the sum of all data, and during this process, the errors were propagated

We continued with the full agonist hGLP‐2(1–33,M10Y) and the partial agonist and competitive antagonist hGLP‐2(3–33,M10Y) for radioligand development using chloramineT for stoichiometric oxidation of the Tyr residue thereby creating [^125^I]‐hGLP‐2(1–33,M10Y) and [^125^I]‐hGLP‐2(3–33,M10Y). To verify the binding properties of the two radioligands, we performed competition binding in cells transiently expressing the hGLP‐2 receptor. Both radioligands showed high‐affinity specific binding for the hGLP‐2 receptor in homologous settings (Figure 2a,b and Table 2), thereby demonstrating successful development of two new radioligands with high and similar binding affinities for the hGLP‐2 receptor. A significantly higher *B*_max_ was found for [^125^I]‐hGLP‐2(3–33,M10Y) (96,6 fmol/10^5^ cells ± 14.9) compared to [^125^I]‐hGLP‐2(1–33,M10Y) (58,0 fmol/10^5^ cells ± 9.2) (Figure 2c). These data are in accordance with a generally higher number of binding sites for GPCR antagonists compared to agonists (Baker & Hill, 2007; Rosenkilde et al., 1994).

**TABLE 2:** Competition binding using the two radioligands

	Ligand	[^125^I]‐hGLP‐2(1–33.M10Y)				[^125^I]‐hGLP‐2(3–33,M10Y)			
		pIC_50_ (M) ± SEM	*K*_i_ (*K*_*D*_) (nM)	% max ± SEM	n	pIC_50_ (M) ± SEM	*K*i (*K*_*D*_) (nM)	% max ± SEM	n
hGLP‐2R	hGLP‐2(1–33)	7.9 ± 0.12	14	*98.8* ± 4.1	3	7.8 ± 0.09	15	*99.4* ± 2.8	5
	hGLP‐2(1–33, M10Y)	7.2 ± 0.07	59 (*K*_*D*_)	*99.7* ± 1.8	5	7.1 ± 0.13	78	*98.9* ± 3.1	6
	hGLP‐2(3–33)	7.2 ± 0.08	57	*100.0* ± 2.1	5	7.1 ± 0.13	81	*98.8* ± 3.1	6
	hGLP‐2(3–33, M10Y)	7.3 ± 0.10	49	*99.5* ± 3.0	5	7.4 ± 0.13	41 (*K*_*D*_)	*98.7* ± 3.6	5
mGLP‐2R	hGLP‐2(1–33)	8.2 ± 0.13	6.6	*97.7* ± 3.3	3	7.7 ± 0.16	18	*92.3* ± 4.1	3
rGLP‐2R	hGLP‐2(1–33)	8.3 ± 0.24	5.4	*89.5* ± 6.2	3	8.2 ± 0.18	6.5	*95.9* ± 5.2	3
hGLP‐1R	hGLP‐2(1–33)	6.9 ± 0.50	130	*37.8* ± 3.6	3	6.5 ± 0.5	330	*26.7* ± 2.3	3
mGLP‐1R	hGLP‐2(1–33)	6.7 ± 0.22	208	*84.5* ± 3.8	3	6.7 ± 0.14	183	*93.3* ± 2.4	3
rGLP‐1R	hGLP‐2(1–33)	N.B.	N.B.	*N.B*.	3	N.B.	N.B.	N.B.	3

*Note*: Competitive binding. Affinities, displayed as pIC_50_, *K*_i_ and *K*_*D*_ values of GLP‐2 and GLP‐2 variants measured in competition with either [^125^I]‐hGLP‐2(1–33,M10Y), left‐hand side, or [^125^I]‐hGLP‐2(3–33,M10Y), right‐hand side. All data were fitted with a three‐parameter logistic curve to obtain pIC_50_. Data represent the mean ± SEM of at least three independent experiments performed in duplicates. The numbers of experiments, *n*, are indicated in the table. N.B. refers to no binding detected. R refers to receptor.

Since ligand‐receptor binding kinetics is considered to be a key determinant of ligand efficacy and onset of action (van der Velden et al., 2020), we determined the association (*k*_on_) and dissociation (*k*_off_) rates, using membranes prepared from cells stably expressing the hGLP‐2 receptor. For both radioligands, the kinetic profiles were best fitted with a one‐phase association and a one‐phase dissociation. Equilibrium of [^125^I]‐hGLP‐2(1–33,M10Y) binding was reached at around 60 min, whereas for [^125^I]‐hGLP‐2(3–33,M10Y), it was reached already at 40 min (Figure 2d). This was also reflected in the observed on‐rate that was 2.7‐fold higher for the latter (*k*_obs_ of 0.074 ± 0.004 min^−1^) compared to the former (*k*_obs_ of 0.027 ± 0.003 min^−1^) (Figure 2f). After reaching equilibrium, the binding was reversed by the addition of 1 μM unlabelled homologous peptide (Figure 2e). Here, [^125^I]‐hGLP‐2(3–33,M10Y) had a 2.0‐fold higher dissociation rate (*k*_off_) than [^125^I]‐hGLP‐2(1–33,M10Y), 0.010 ± 0.001 min^−1^ and 0.005 ± 0.001 min^−1^, respectively (Figure 2f). The calculated on‐rate (association rate constant, *k*_on_) was consequently 2.7‐fold higher for the antagonist (0.308 ± 0.028 nM^−1^*min^−1^), compared to the agonist (0.115 ± 0.019 nM^−1^ min^−1^) (Figure 2f). Thus, the receptor association as well as the dissociation is faster for the antagonist compared to the full agonist. Finally, we calculated the *K*_*D*_ values from the kinetic profiles to be 0.046 ± 0.013 nM and 0.032 ± 0.005 nM for hGLP‐2(1–33,M10Y) and hGLP‐2(3–33,M10Y), respectively.

### 3.3 Native hGLP‐2(1–33) binds to the hGLP‐2 receptor with highest affinity

**FIGURE 3:**
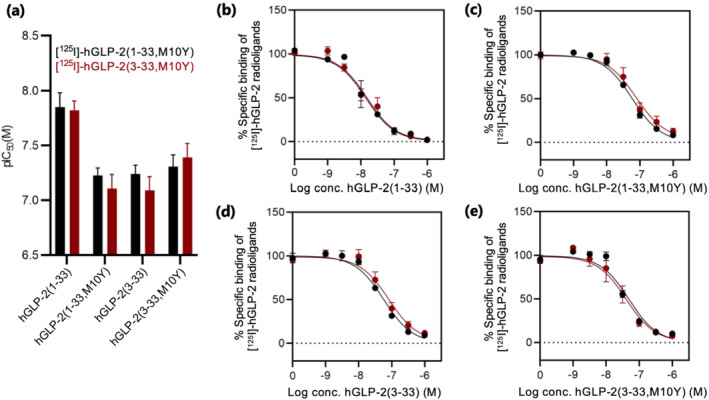
Heterologous competition binding using radiolabelled hGLP‐2(1–33,M10Y) and hGLP‐2(3–33,M10Y). (a) Bar chart of the pIC_50_ values for binding of [^125^I]‐hGLP‐2(1–33,M10Y) (black) and [^125^I]‐hGLP‐2(3–33,M10Y) (red). (b–e) Competition binding of [^125^I]‐hGLP‐2(1–33,M10Y) (black) and [^125^I]‐hGLP‐2(3–33,M10Y) (red) displaced by increasing concentrations of (b) hGLP‐2(1–33) (*n* = 5), (c) hGLP‐2(1–33,M10Y) (*n* = 5 for [^125^I]‐hGLP‐2(1–33,M10Y) and *n* = 6 for [^125^I]‐hGLP‐2(3–33,M10Y)), (d) hGLP‐2(3–33) (*n* = 5 for [^125^I]‐hGLP‐2(1–33,M10Y) and *n* = 6 for [^125^I]‐hGLP‐2(3–33,M10Y)) and (e) hGLP‐2(3–33,M10Y) (*n* = 5). To compensate for inter‐assay variations data were normalized to the specific binding of each ligand, against each of the radioligands, at the hGLP‐2 receptor within each assay

In order to determine whether agonists and antagonists competed equally for binding to the hGLP‐2 receptor, we measured heterologous binding by displacing each of the two novel GLP‐2 radioligands with the four unlabelled peptides; hGLP‐2(1–33), hGLP‐2(3–33), hGLP‐2(1–33,M10Y) and hGLP‐2(3–33,M10Y) (Figure 3). Overall, all four ligands were able to compete with both radioligands, with no major differences in their binding affinities whether using the agonist or the antagonist radioligand (Figure 3a and Table 2). However, native hGLP‐2(1–33) did have a four‐ to fivefold higher affinity compared to the other three hGLP‐2 variants (Figure 3 and Table 2). The decreased affinity of hGLP‐2(1–33,M10Y) compared to hGLP‐2(1–33) is consistent with its decreased potency in cAMP accumulation as well as β‐arrestin recruitment activity assays (Figure 1b–d).

### 3.4 Selectively binding profile of the two radioligands for related class B1 GPCRs

**FIGURE 4:**
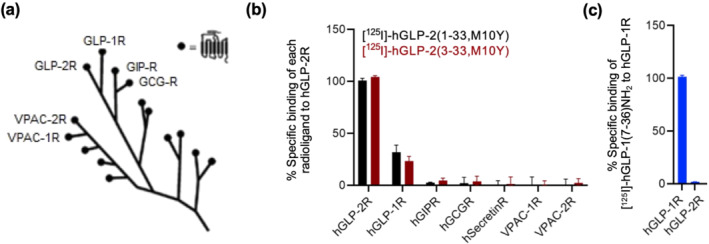
Exploratory data. Test for selectivity among class B1 GPCRs (exploratory data). (a) Phylogenetic tree of the class B1 subfamily GPCRs consisting of the GLP‐2 receptor (R) and 14 sequence related GPCRs (modified from Gasbjerg et al., 2018). (b) Heterologous binding of [^125^I]‐hGLP‐2(1–33,M10Y) (black) and [^125^I]‐hGLP‐2(3–33,M10Y) (red) to the hGLP‐1 receptor (*n* = 3), hGIP receptor (*n* = 2), hglucagon receptor (*n* = 2), hSecretin receptor (*n* = 2), VPAC_1_ receptor (*n* = 2) and VPAC_2_ receptor (*n* = 2) displaced by increased concentrations of hGLP‐2(1–33). (c) Homologous competition binding of [^125^I]‐GLP‐1(7–36) to the hGLP‐2 receptor (*n* = 3) and hGLP‐1 receptor (positive control) (*n* = 3) displaced by increasing concentration of GLP‐1(7–36). To compensate for inter‐assay variations, data were normalized to the specific binding of (b) hGLP‐2 receptor or (c) hGLP‐1 recepetor for each individual radioligand within each assay

Given the high sequence similarity between class B1 receptors and their peptide ligands, we next determined whether the two radioligands cross‐reacted with the closely related class B1 receptors hGIP receptor, hGLP‐1 receptor, hglucagon, hsecretin receptor, hVPAC_1_ receptor and hVPAC_2_ receptor (Figure 4a). While we observed no specific binding for five of the six receptors, a low but significant binding was observed for both radioligands to the hGLP‐1 receptor (Figures 4b and S1). This cross‐reaction intrigued us to test the opposite pairing with binding of [^125^I]‐hGLP‐1(7–36) to the hGLP‐2 receptor, which turned out to be undetectable (Figure 4c). In addition to GLP‐2(1–33) and GLP‐2(3–33) identified in our study, a broad range of other peptides are known to bind to the GLP‐1 receptor (glucagon, oxyntomodulin, besides GLP‐1 receptor) (Holst et al., 2018; Jorgensen et al., 2007; Skov‐Jeppesen et al., 2019). In contrast, this broad specificity in binding does not seems to be the case for the GLP‐2 receptor, which seemingly exhibits a narrower binding of only GLP‐2‐based ligands.

### 3.5 Endogenous hGLP‐2(1–33) and hGLP‐2(1–33,M10Y) also activate the hGLP‐1 receptor, but do not induce arrestin recruitment to this receptor

**FIGURE 5:**
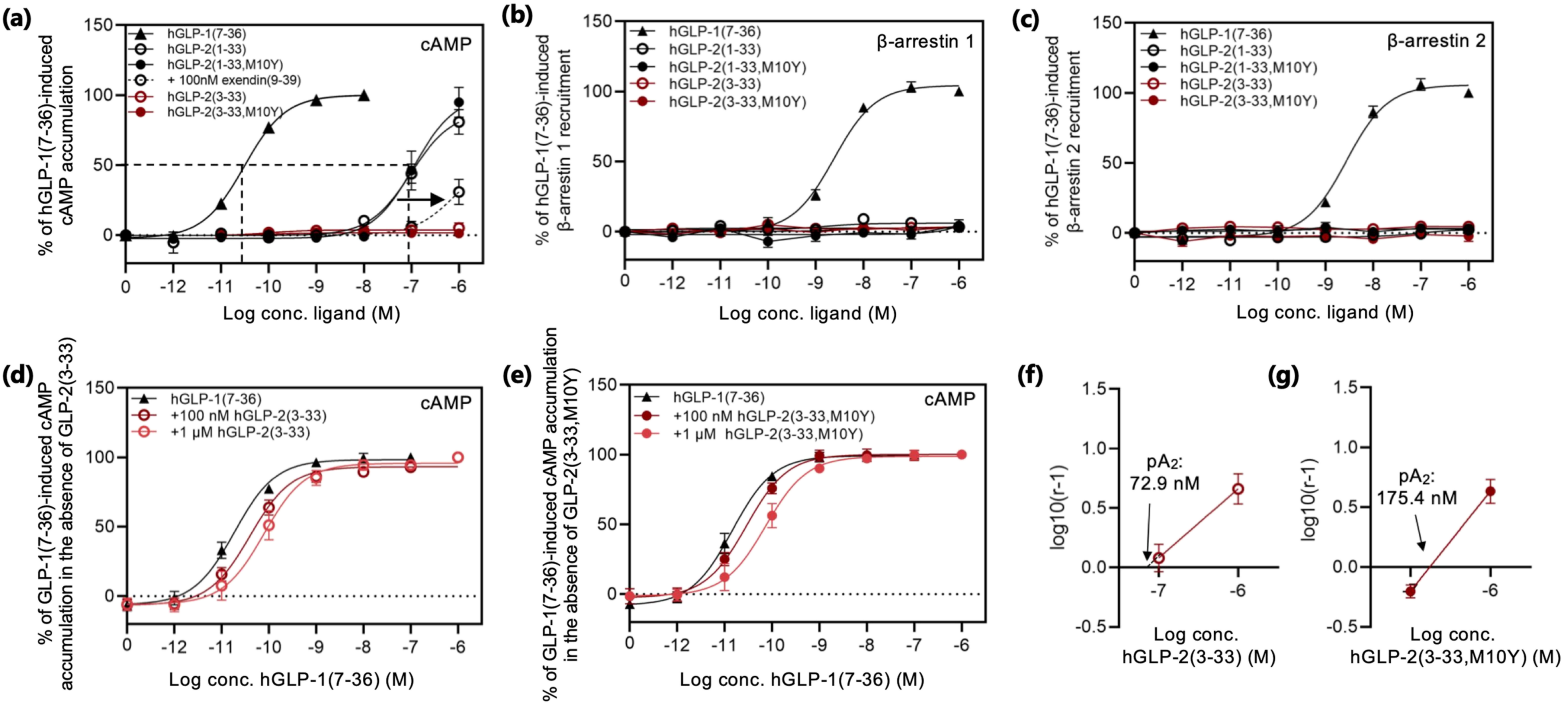
Activity of the hGLP‐2 variants at the hGLP‐1 receptor. Dose–response curves in (a) cAMP accumulation, (b) β‐arrestin 1 recruitment and (c) β‐arrestin 2 recruitment for the hGLP‐1 receptor stimulated with increasing concentrations of hGLP‐1(7–36), hGLP‐2(1–33), hGLP‐2(1–33,M10Y) (plus/minus 100 nM exendin(9–39) in (a)), all in black, hGLP‐2(3–33) and hGLP‐2(3–33,M10Y), both in red. cAMP accumulation dose–response curves for hGLP‐1(7–36) in the presence of 100 nM and 1 μM (d) hGLP‐2(3–33) or (e) hGLP‐2(3–33,M10Y) and the corresponding Schild plots shown in (f) hGLP‐2(3–33) and (g) hGLP‐2(3–33,M10Y) with pA_2_ values. To compensate for inter‐assay variations, data were normalized to hGLP‐1(7–36) within each experiment

The binding of the GLP‐2 peptides to the hGLP‐1 receptor inspired us to further characterize the action of hGLP‐2 in the hGLP‐1 receptor system using the same experimental setup as for the hGLP‐2 receptor (Figure 1b–d). Both full‐length peptides, hGLP‐2(1–33) and hGLP‐2(1–33,M10Y), activated the hGLP‐1 receptor yet with >3,000‐fold lower potency compared to GLP‐1 (Figure 5a and Table 1). In contrast, no activation was observed for the two N terminally truncated variants hGLP‐2(3–33) and hGLP‐2(3–33,M10Y) (Figure 5a and Table 1). To further describe the hGLP‐2 mediated activation of the GLP‐1 receptor, we reversed the signal by employing the competitive GLP‐1 receptor antagonist exendin(9‐39) (Schirra et al., 1998). A rightward shift was observed for the dose–response curve of hGLP‐2(1–33) in the presence of exendin(9–39) (Figure 5a and Table 1), suggesting that the cAMP accumulation induced by hGLP‐2(1–33) was mediated through the hGLP‐1 receptor, in a similar manner, as that of GLP‐1. With regards to β‐arrestin 1 and 2 recruitment, neither of the GLP‐2 variants induced any activation, whereas a strong recruitment of both arrestins was induced by hGLP‐1(7–36) (Figure 5b,c). We also tested for antagonistic properties of the N terminally truncated variants with respect to cAMP accumulation. Here, increasing concentration (100 nM and 1 μM of hGLP‐2(3–33) or hGLP‐2(3–33,M10Y) resulted in a slight rightward shift of the dose–response curve of hGLP‐1 (2.1‐ and 4.2‐fold for hGLP‐2(3–33) and 1.9‐ and 5.1‐fold for hGLP‐2(3–33,M10Y)) (Figure 5d,e and Table 1). The corresponding Schild plot analyses revealed competitive nature of both antagonists on the hGLP‐1 receptor with pA2 values of 72.9 and 175.4 nM, respectively (Figure 5f,g).

### 3.6 Autoradiography and immunohistochemistry in mice reveal GLP‐2 receptor expression in sub‐epithelial myofibroblasts (SEMF)

**FIGURE 6:**
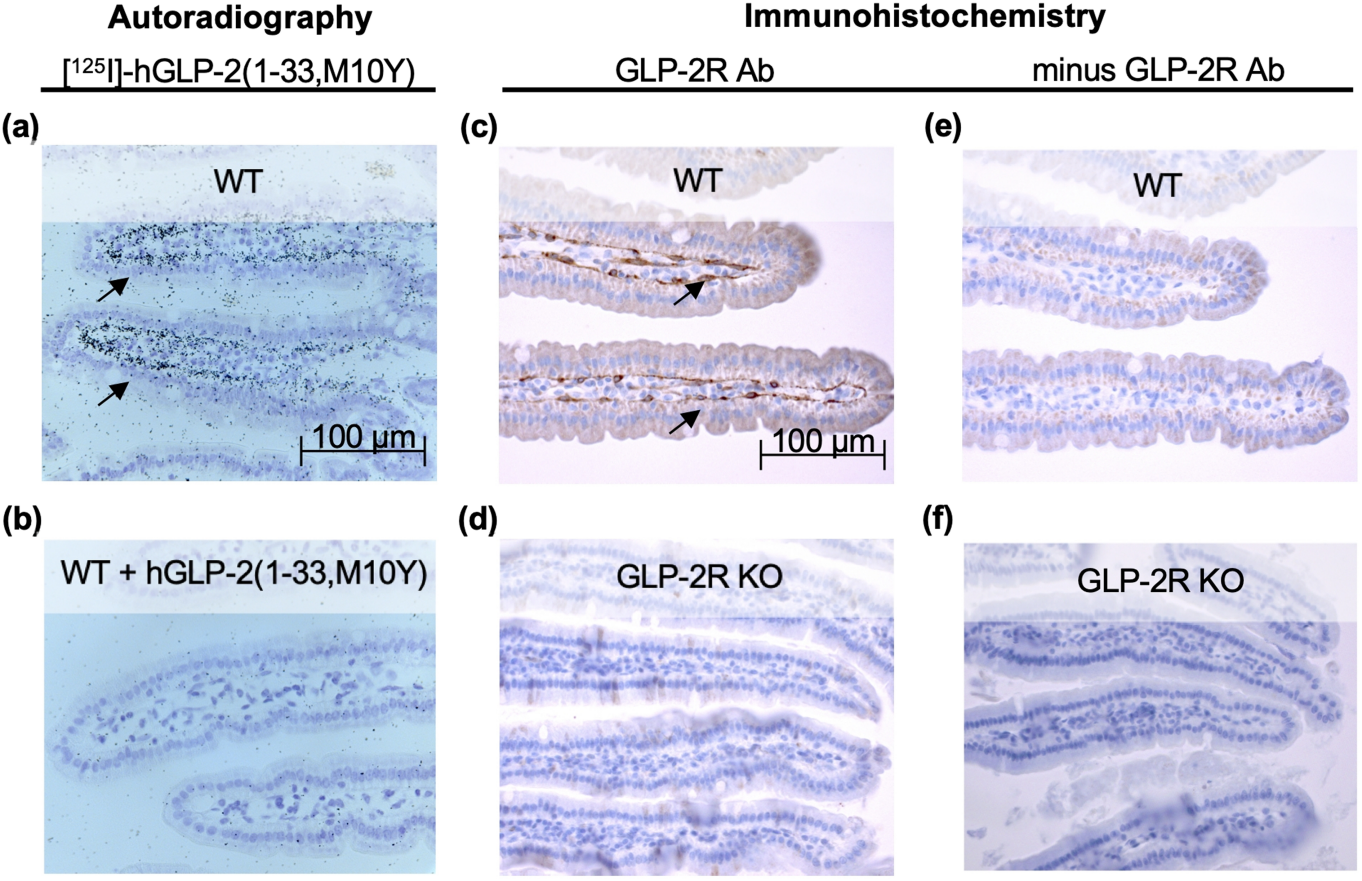
Autoradiography and immunohistochemistry in mice. Histological sections of the small intestine after (a, b) autoradiography in mice injected with [^125^I]‐hGLP‐2(1–33,M10Y) for (a) wild type (WT) mice and (b) WT mice pre‐injected with >10,000‐fold excess unlabelled hGLP‐2(1–33,M10Y) and (c, f) immunohistochemistry using a polyclonal rabbit GLP‐2 receptor (R) antibody in (c) WT mice and (d) GLP‐2 receptor KO mice. In the absence of the primary GLP‐2 receptor antibody, the secondary biotinylated goat‐anti rabbit antibody revealed no staining of either (e) WT or (f) GLP‐2 receptor KO mice. Scale bar 100 μm

After validating the binding of [^125^I]‐hGLP‐2(1–33,M10Y) to mouse and rat GLP‐2 receptors by competition binding experiments (Figure S2), we continued with autoradiography studies in mice. In all mice examined, we observed strong labelling in the SEMFs of the GI tract (Figures 6a and S3a–c). Injection of 30 nmol unlabelled GLP‐2(1–33) prior to the radioligand abrogated labelling (Figures 6b and S3d–f), supporting the specificity of binding by [^125^I]‐hGLP‐2(1–33,M10Y). To confirm that the binding to SEMFs was indeed driven by GLP‐2 receptor binding, we performed immunohistochemistry in WT and GLP‐2 receptor KO mice using a previously described GLP‐2 receptor antibody (Ørskov et al., 2005). Consistent with the autoradiography, we observed strong staining of the SEMFs in the WT mice (Figures 6c and S3g–i), while no staining was observed in similar tissue from the GLP‐2 receptor KO mice (Figures 6d and S3j–l). Furthermore, no staining was observed in the WT and GLP‐2 receptor KO mice without the GLP‐2 specific antibody (Figure 6e,f). These data confirm GLP‐2 receptor expression at the protein level, thereby strengthening what was previously shown at the level of GLP‐2 receptor mRNA (El‐Jamal et al., 2014; Ørskov et al., 2020; Yusta et al., 2000; Yusta et al., 2019). We also observed labelling by [^125^I]‐hGLP‐2(1–33,M10Y) in pancreatic islet cells that was abrogated in the presence of unlabelled GLP‐2(1–33) (Figure S4a–c and d–f, respectively). This pancreatic staining was not detectable by the GLP‐2 receptor‐specific antibody in WT mice, suggesting that it was most likely due to binding to the GLP‐1 receptor expressed at high levels in the islets (Figure S1g–l). In conjunction with the binding data, these data demonstrate dual selectivity of the radioligands with high affinity to the GLP‐2 receptor and low affinity to the GLP‐1 receptor.

## 4 DISCUSSION

Until recently, very little structural information was available of the hGLP‐2 receptor, despite emerging evidence for a biological importance of GLP‐2 as a trophic hormone for the gut and bones. An increasing number of high‐resolution structures of class B1 GPCRs have recently been published (Smit et al., 2021; Wu et al., 2020; Zhang et al., 2017), and in December 2020, a structure of the GLP‐2 receptor was presented (Sun et al., 2020). Prior to this, a handful of studies had however elucidated on the structural requirements for GLP‐2's interaction with its receptor. In 2000, DaCambra et al. (2000) performed an alanine (Ala)‐scan within the DPP‐4 resistant h[Gly2]GLP‐2(1–33) peptide and showed reduced receptor activation (cAMP accumulation) of the rGLP‐2 receptor upon alterations in the N‐terminal part of the peptide. Here, Ala replacement of [His1] and [Asp3] of hGLP‐2 severely reduced receptor activation with only modest changes in binding affinity. These data demonstrate the importance of the GLP‐2 N‐terminus for receptor activation, as also illustrated by the partial agonism of the competitive antagonist GLP‐2(3–33) (Thulesen et al., 2002) (Figure 1). In contrast, Ala‐scan within the C‐terminal region of h[Gly2]GLP‐2 revealed severely reduced affinities demonstrating a central role of the C‐terminal part for receptor binding (DaCambra et al., 2000).

In 2011, Venneti and Hewage (2011) presented the first three‐dimensional solution structure of GLP‐2 by NMR. This structure supported the distinct roles of the N‐ and C‐terminal parts of GLP‐2 and revealed a stable alpha‐helical conformation of the central region (between [Phe6] and [Ile27]) and a less well‐defined helical conformation in the C‐terminal region. The binding interface with the extracellular domain of the receptor was predicted to be between [Leu17] and [Lys30], while the N‐terminal part of GLP‐2 from [His1] to [Asp16] lacked contact with the extracellular domains of the GLP‐2 receptor. The central and distinct roles of the N‐ and C‐terminal parts of GLP‐2 in, respectively, receptor activation and receptor binding, were supported by Yamazaki et al. (2013), showing a decreased intrinsic placental alkaline phosphatase activity (driven by cAMP) for GLP‐2(3–33), (6–33) and (11 to 13–33). More recently, Wiśniewski et al. (2016) replaced each residue in the DPP‐4 resistant [Gly2,Nle10]hGLP‐2(1–30) analogue with its D‐enantiomer in a systematic approach to gain insight into the GLP‐2 receptor recognition revealing a loss of potency at positions 5, 8, 9, 12 and 14 in the N‐terminus, and similar loss for position 17–20, 25 and 29. Consistent with this, the C‐terminus of GLP‐2 orientates towards a hydrophilic cavity in the NMR structure (Venneti & Hewage, 2011). The newly presented GLP‐2 receptor structure, solved by cryo‐EM, confirms the previously suggested ligand‐binding interaction and reveals overall similar binding modes of the endogenous peptide ligands in class B1 GPCRs (Liang, Khoshouei, Glukhova, et al., 2018; Qiao et al., 2020; Sun et al., 2020; Wu et al., 2020; Zhang et al., 2017, 2018; Zhao et al., 2019).

We found that the M10Y‐modification in hGLP‐2(1–33) had a minor impairment on the binding affinity and potency in cAMP accumulation, and a larger effect on β‐arrestin recruitment as both potencies and efficacies were affected on β‐arrestin 1 as well as β‐arrestin 2 recruitment. This could reflect the general weaker arrestin recruitment compared to Gα_s_ coupling of class B1 receptors as described for the GIP receptor (Gabe et al., 2018) and the GLP‐1 receptor (van der Velden et al., 2021). Accordingly, we observed a 7.5‐ to 16‐fold lower potency of the endogenous agonist hGLP‐2(1–33) in arrestin recruitment versus cAMP accumulation. According to the NMR structure, Met10 is positioned at the beginning of the alpha‐helix and is not directly part of the binding interface of the GLP‐2 receptor (Venneti & Hewage, 2011). Consistent with this, Wiśniewski et al. (2016) replaced the oxidation and alkylation‐prone Met residue at position 10 of hGLP‐2 by the isosteric norleucine (Nle) showing that this Met residue is dispensable for the function of GLP‐2. Met is characterized by a sulfur atom in the side chain, which is highly sensitive to reactive oxygen species (ROS) that often changes structural and functional properties of proteins (Black & Mould, 1991; Kim et al., 2014). ROS‐mediated oxidation occurs by the addition of a single oxygen molecule to the sulfur atom, forming methionine sulfoxide (MetSO) (Kim et al., 2014), which creates a chiral centre around the sulfur atom and overall results in a more rigid and more polar side chain compared to the unoxidized Met residue (Black & Mould, 1991). These changes can have profound structural and functional consequences (Gu et al., 2015; Hoshi & Heinemann, 2001). To protect for oxidative damage of the Met in GLP‐2 during the oxidative iodination, and since Met is dispensable for GLP‐2 function (Drucker et al., 1996; Venneti & Hewage, 2011; Wiśniewski et al., 2016; Yamazaki et al., 2013), we replaced Met10 with a Tyr residue. Thereby, we created a target site for oxidative iodination using [^125^I] in the full agonist (GLP‐2(1–33)) and the metabolite (GLP‐2(3–33)). These modifications created the two peptides: hGLP‐2(1–33,M10Y) and hGLP‐2(3–33,M10Y), which allowed us to investigate the binding of both a full agonist [^125^I]‐hGLP‐2(1–33,M10Y) and an antagonist with low intrinsic activity (i.e. partial agonist) [^125^I]‐hGLP‐2(3–33,M10Y) to a class B1 GPCR.

With these two radioligands, we were in a unique position, for the first time among class B1 GPCRs, to describe the binding kinetics of two structurally similar, but functionally different peptides. Our studies showed that the full agonist displayed the slowest on‐ and off‐rate of the two. This is relevant as binding parameters, *k*_on_ and *k*_off_ and residence time (i.e. 1/*k*_off_), have been emphasized as important in describing a ligand's *in vivo* efficacy as well as the onset of action (van der Velden et al., 2020). The slower on‐rate and off‐rate for the agonist could reflect a more complex binding compared to the antagonist in line with expected induction of active receptor states. However, for all four implemented ligands, we observed rather similar affinities irrespective of choice of radioligand, suggesting that the receptor easily interchanges between conformations induced by the two radioligands. The lower K_*D*_ values calculated from the kinetic parameters (*K*_*D*_ = *k*_off_/*k*_on_) compared to those from the competition binding (*K*_*D*_ = IC_50_‐[L]) likely reflect the underlying experimental differences (HEK‐293 membranes stably expressing GLP‐2 receptor versus whole cell binding on COS‐7 transiently expressing the receptor, 30°C versus 4°C, and indirect radioactive measurement using a SPA readout versus direct gamma radiation), and may also be ascribed to the nature of the calculations and the assumptions behind their use. The *K*_*D*_ value determined from the competition binding experiments is an estimated value using the Cheng and Prusoff equation where several assumptions and experimental conditions are needed for the use of this equations (DeBlasi et al., 1989), while the *K*_*D*_ obtained from kinetic experiments is directly calculated from on, and off‐rates.

The specific tissue expression of the GLP‐2 receptor remains controversial. It has been reported that the GLP‐2 receptor mRNA transcript and protein are expressed in SEMFs (El‐Jamal et al., 2014; Ørskov et al., 2005). Here, we confirm receptor expression at the protein level in SEMFs in the intestine of mice by using autoradiography with [^125^I]‐hGLP‐2(1–33,M10Y). Moreover, the immunohistochemistry using the GLP‐2 receptor‐specific antibody confirmed this location, as no staining was observed in the intestine from GLP‐2 receptor KO mice. In contrast, the observed labelling in the pancreatic islet cells by autoradiography was not confirmed by the GLP‐2 receptor antibody, thereby suggesting that pancreatic labelling is due to the binding of [^125^I]‐hGLP‐2(1–33,M10Y) to islet cells expressing high levels of the GLP‐1 receptor, as also supported by the dual selectivity of this radioligand with binding to the human and mouse GLP‐1 receptor. Promiscuity is known within class B1 GPCRs, demonstrated by the activation of the GIP receptor by GLP‐2 (Skov‐Jeppesen et al., 2019), the binding and activation of both the GLP‐1 receptor and the glucagon receptor by oxyntomodulin (Holst et al., 2018; Jorgensen et al., 2007) and the activation of the GLP‐1 receptor by glucagon (Svendsen et al., 2018). Thus, cross‐activation is a common phenomenon within class B1 GPCRs, which is reflected in the high sequence similarities observed among the receptors and across species. For rodent GLP‐2 receptors, 81% and 79% sequence identities are found for the mGLP‐2 receptor and rGLP‐2 receptor to the hGLP‐2 receptor, respectively, explaining the high‐affinity binding observed for both radioligands to the rodent GLP‐2 receptors.

In conclusion, we developed two new radioligands for the GLP‐2 receptor; both with high affinity to the human, rat and mouse GLP‐2 receptor, and with low affinity for the mouse and human GLP‐1 receptor. With these, we show differential binding kinetics of full agonist and partial agonist with antagonistic properties to the GLP‐2 receptor and confirm GLP‐2 receptor expression at the protein level in the GI tract's subepithelial myofibroblasts. Our observations are of importance for tissue localization and structural characterization for not only the GLP‐2 receptor, but also for other class B1 GPCRs.

## AUTHOR CONTRIBUTIONS

The authors' contributions are as follows: conceptualization: Mette M. Rosenkilde designed and supervised the experiments. Sarina Gadgaard, Wijnand J. C. van der Velden, Sine P Schiellerup, Jenna Elizabeth Hunt, Maria B. N. Gabe, Johanne Agerlin Windeløv, Geke Aline Boer, Hannelouise Kissow and Cathrine Ørskov performed the experiments, data analysis and prepared the figures within coordination with Sarina Gadgaard. Cathrine Ørskov, Jens J. Holst and Bolette Hartmann and Mette M. Rosenkilde provided intellectual content.

## CONFLICT OF INTEREST

The authors declare that the research was conducted in the absence of any commercial or financial relationships that could be construed as a potential conflict of interest. M.M.R, J.J.H and B. H are founders of Bainan Biotech but declare that the research was conducted in the absence of any commercial or financial relationships.

## DECLARATION OF TRANSPARENCY AND SCIENTIFIC REGOUR

This declaration acknowledges that this paper adheres to the principles for transparent reporting and scientific rigour of preclinical research as stated in the *BJP* guidelines for Design and Analysis, Immunoblotting and Immunochemistry and Animal Experimentation, and as recommended by funding agencies, publishers and other organizations engaged with supporting research.

## Supporting information


**Figure S1.** Exploratory data. Test for selectivity among class B1 GPCRs. Competition binding curves of [^125^I]‐hGLP‐2(1–33,M10Y) (black) and [125I]‐hGLP‐2(3–33,M10Y) (red) to (a) hGLP‐1 receptor (R) (n = 3), (b) hGIP receptor (n = 2), (c) hglucagon (GCG) receptor (n = 2), (d) hsecretin receptor (n = 2), I VPAC_1_ receptor (n = 2), and (f) VPAC_2_ receptor (n = 2) displaced by increasing concentrations of hGLP‐2(1–33). To compensate for inter‐assay variations data have been normalized for each individual radioligand to the hGLP‐2 receptor within each assay.

**Figure S2.** Exploratory data. Binding of the two radioligands to rodent GLP‐2 receptors and GLP‐1 receptors. Competition binding of [^125^I]‐hGLP‐2(1–33,M10Y) (black) and [^125^I]‐hGLP‐2(3–33,M10Y) (red) to (a) the mGLP‐2 receptor (n = 3), (b) the rGLP‐2 receptor (n = 3), (c) mGLP‐1 receptor (n = 3), and (d) rGLP‐1 receptor (n = 3). To compensate for inter‐assay variations data were normalized to the specific binding of respectively mGLP‐2 receptor and rGLP‐2 receptor for each individual radioligand within each assay.

**Figure S3.** Autoradiography and immunohistochemistry in mice intestine. Histological sections of the small intestine after (a‐f) autoradiography in mice injected with [^125^I]‐hGLP‐2(1–33,M10Y) for (a‐c) WT mice and (d‐f) WT mice pre‐injected with unlabeled hGLP‐2(1–33,M10Y), and (g‐l) immunohistochemistry using a GLP‐2 receptor antibody in (g‐i) WT mice and (j‐l) GLP‐2 receptor KO mice. The histological sections were counterstained with haematoxylin. Scale bar 100 μm.

**Figure S4.** Autoradiography and immunohistochemistry in mice pancreatic islet cells. Histological sections of the pancreatic islet cells after (a‐f) autoradiography in mice injected with [^125^I]‐hGLP‐2(1–33,M10Y) for (a‐c) WT mice and (d‐f) WT mice pre‐injected with unlabeled hGLP‐2(1–33,M10Y), and (g‐i) immunohistochemistry using a GLP‐2 receptor antibody in (g‐i) WT mice and (j‐l) GLP‐2 receptor KO mice. The histological sections were counterstained with haematoxylin. Scale bar 100 μm.

## Data Availability

The data that support the findings of this study are available from the corresponding author upon reasonable request. Some data may not be made available because of privacy or ethical restrictions.
