# Supplementary figures and images for: Novel agonist and antagonist radioligands for the GLP‐2 receptor. Useful tools for studies of basic GLP‐2 receptor pharmacology
Source: Br J Pharmacol. 2022 Jan 11;179(9):1998–2015. doi: 10.1111/bph.15766 (PMC9303331; doi:10.1111/bph.15766)

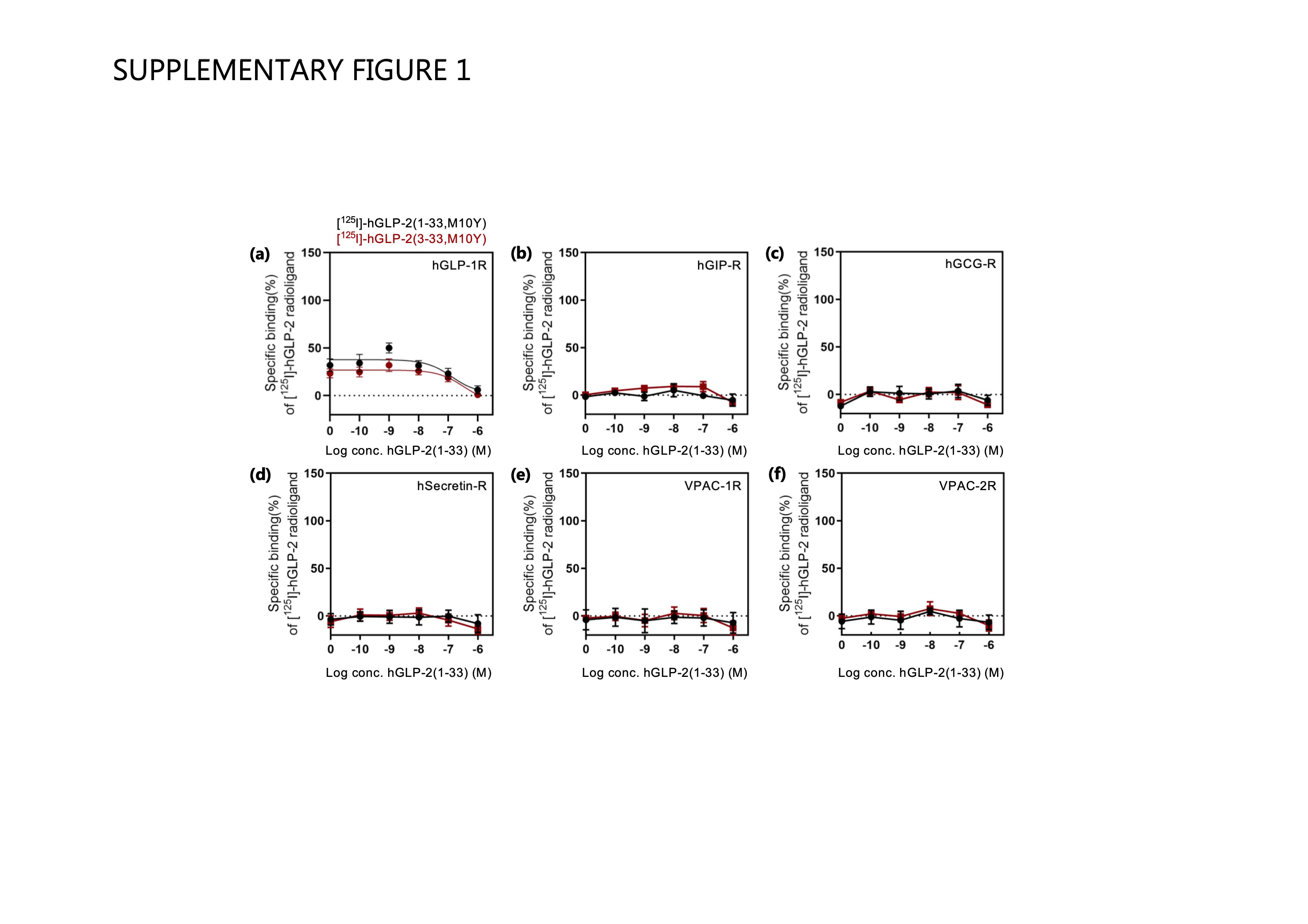

Supplement: Supplementary file 1 — Figure S1. Exploratory data. Test for selectivity among class B1 GPCRs. Competition binding curves of [125I]‐hGLP‐2(1–33,M10Y) (black) and [125I]‐hGLP‐2(3–33,M10Y) (red) to (a) hGLP‐1 receptor (R) (n = 3), (b) hGIP receptor (n = 2), (c) hglucagon (GCG) receptor (n = 2), (d) hsecretin receptor (n = 2), I VPAC1 receptor (n = 2), and (f) VPAC2 receptor (n = 2) displaced by increasing concentrations of hGLP‐2(1–33). To compensate for inter‐assay variations data have been normalized for each individual radioligand to the hGLP‐2 receptor within each assay. [file BPH-179-1998-s003.jpg]

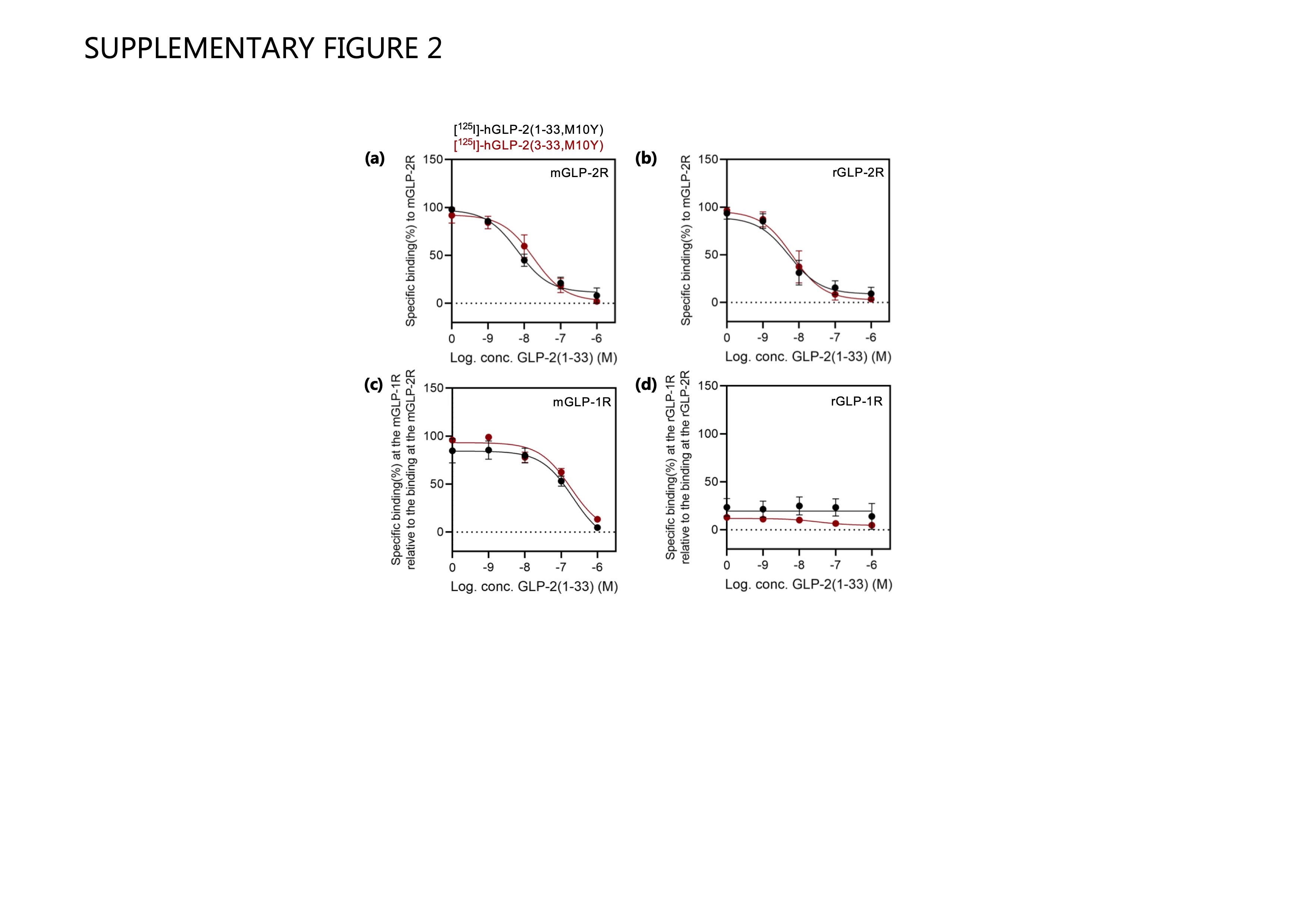

Supplement: Supplementary file 2 — Figure S2. Exploratory data. Binding of the two radioligands to rodent GLP‐2 receptors and GLP‐1 receptors. Competition binding of [125I]‐hGLP‐2(1–33,M10Y) (black) and [125I]‐hGLP‐2(3–33,M10Y) (red) to (a) the mGLP‐2 receptor (n = 3), (b) the rGLP‐2 receptor (n = 3), (c) mGLP‐1 receptor (n = 3), and (d) rGLP‐1 receptor (n = 3). To compensate for inter‐assay variations data were normalized to the specific binding of respectively mGLP‐2 receptor and rGLP‐2 receptor for each individual radioligand within each assay. [file BPH-179-1998-s004.jpg]

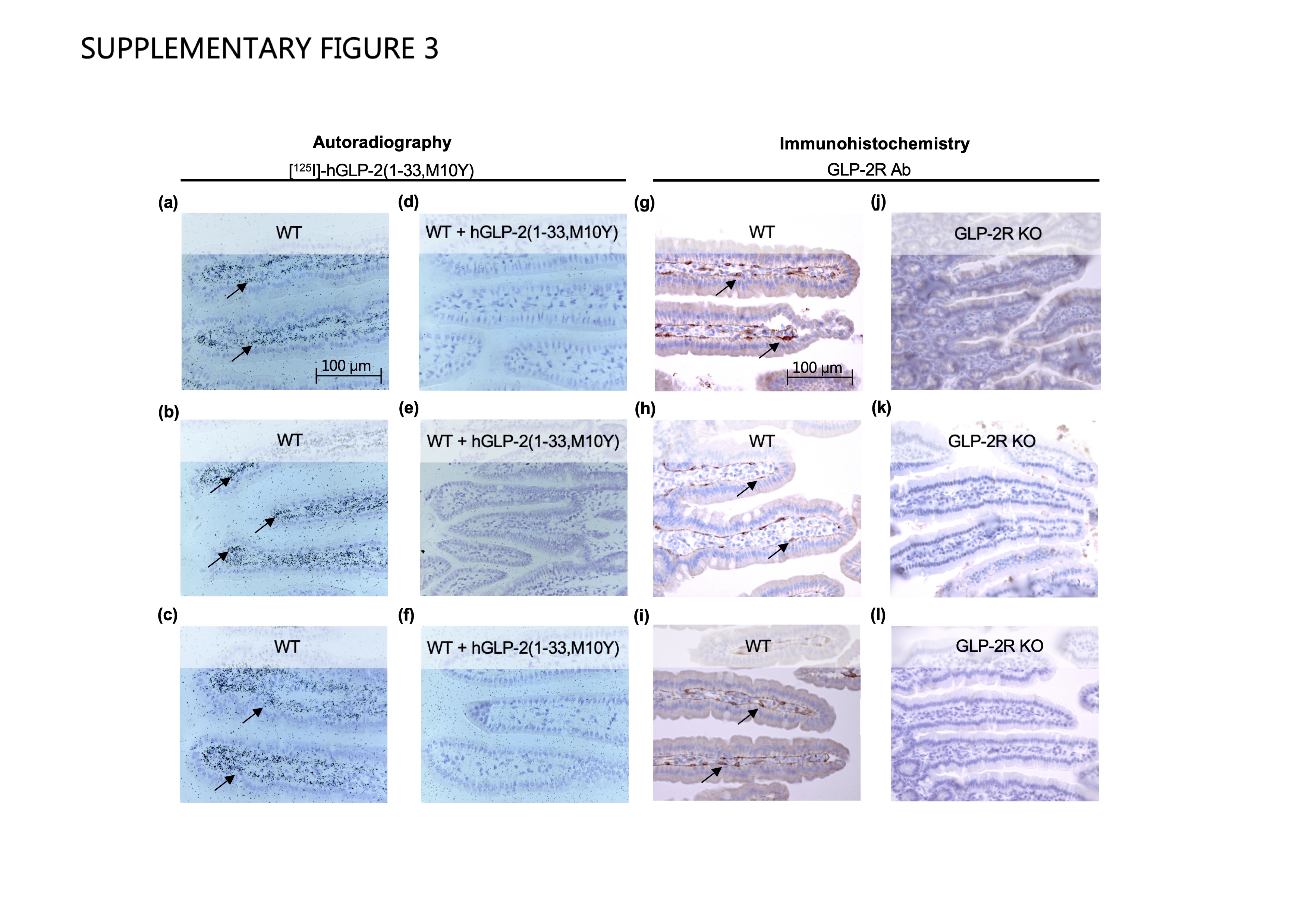

Supplement: Supplementary file 3 — Figure S3. Autoradiography and immunohistochemistry in mice intestine. Histological sections of the small intestine after (a‐f) autoradiography in mice injected with [125I]‐hGLP‐2(1–33,M10Y) for (a‐c) WT mice and (d‐f) WT mice pre‐injected with unlabeled hGLP‐2(1–33,M10Y), and (g‐l) immunohistochemistry using a GLP‐2 receptor antibody in (g‐i) WT mice and (j‐l) GLP‐2 receptor KO mice. The histological sections were counterstained with haematoxylin. Scale bar 100 μm. [file BPH-179-1998-s002.jpg]

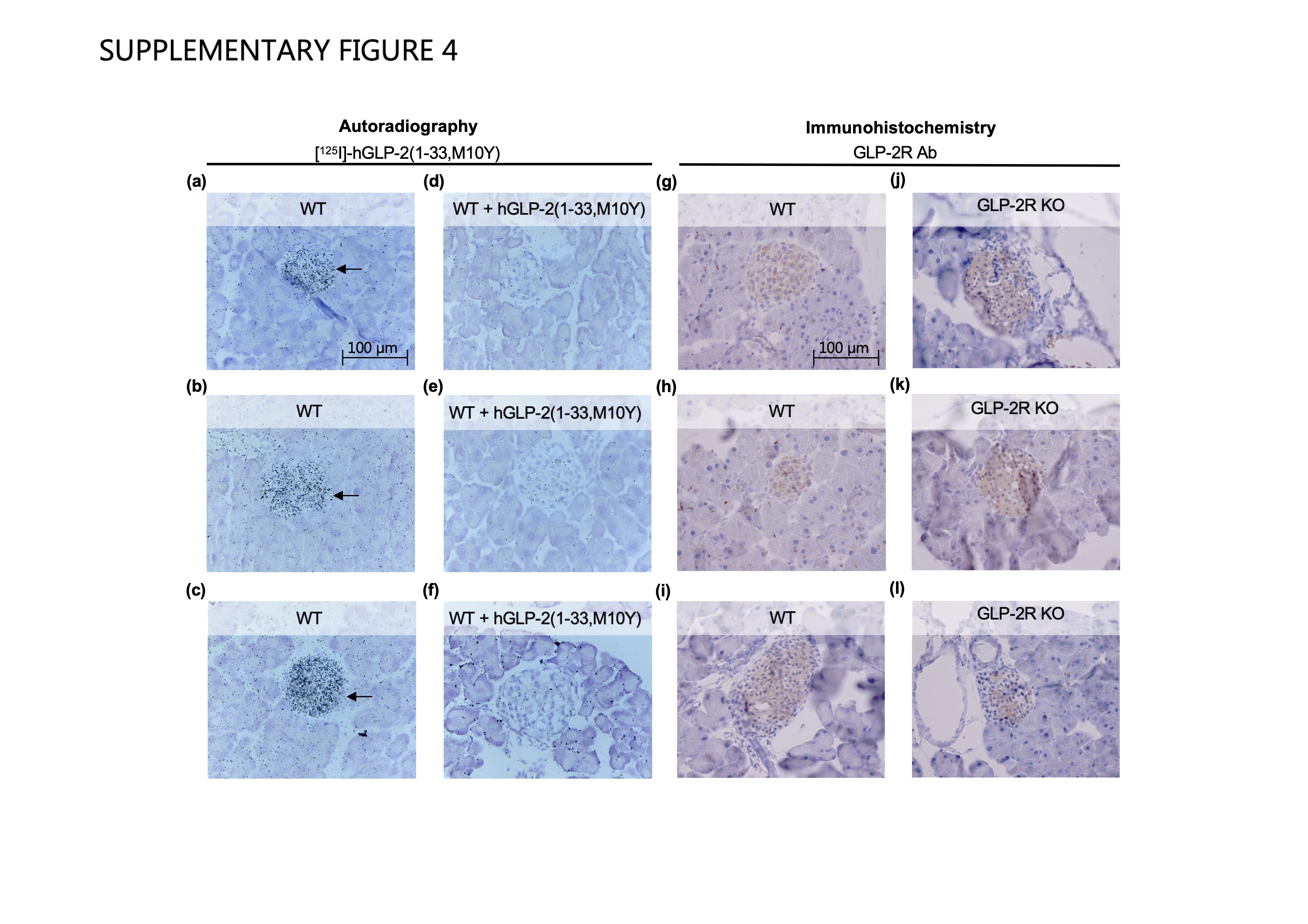

Supplement: Supplementary file 4 — Figure S4. Autoradiography and immunohistochemistry in mice pancreatic islet cells. Histological sections of the pancreatic islet cells after (a‐f) autoradiography in mice injected with [125I]‐hGLP‐2(1–33,M10Y) for (a‐c) WT mice and (d‐f) WT mice pre‐injected with unlabeled hGLP‐2(1–33,M10Y), and (g‐i) immunohistochemistry using a GLP‐2 receptor antibody in (g‐i) WT mice and (j‐l) GLP‐2 receptor KO mice. The histological sections were counterstained with haematoxylin. Scale bar 100 μm. [file BPH-179-1998-s001.jpg]
